# Sustained Effects of Physiotherapy Interventions on Balance, Gait, and General Motor Function in Patients with Parkinson’s Disease: A Systematic Review and Meta-Analysis

**DOI:** 10.3390/neurosci7020042

**Published:** 2026-04-03

**Authors:** Madela Hasani, Ilektra Sidiropoulou, Anna Christakou, Antonia Marazioti, Spyridon Konitsiotis, Epameinondas Lyros

**Affiliations:** 1 Department of Physiotherapy, School of Health Sciences, University of Peloponnese, 23100 Sparta, Greece; madelahasani@gmail.com (M.H.); ilektrasidiropouloy@gmail.com (I.S.); a.christakou@go.uop.gr (A.C.); a.marazioti@uop.gr (A.M.); 2 Department of Neurology, Medical School, University of Ioannina, 45110 Ioannina, Greece; skonitso@gmail.com

**Keywords:** Parkinson’s-disease, physiotherapy, rehabilitation, balance, gait, motor function

## Abstract

Background and purpose: Balance and gait problems pose a significant burden in Parkinson’s disease (PD), and they are often poorly treated with levodopa. We intended to summarize evidence of mid- and long-term impact of various physiotherapeutic interventions (≥3 months post-intervention) on dynamic balance, gait, and general motor function in patients with PD. Method: A systematic search was conducted across the PubMed, Cochrane Library, and Scopus databases to identify controlled clinical trials on sustained effects of various exercise interventions in PD on the outcomes of interest (lasting ≥ 3 months after completion of the exercise program). We conducted meta-analyses on commonly used clinical measures of dynamic balance and gait ability, as well as on UPDRS-III scores using the Comprehensive Meta-Analysis Software (CMA). Results: A total of 26 studies were included in meta-analyses, with a total of 1261 participants in the experimental and 989 participants in the control groups. Positive cumulative effects at the post-exercise follow-up (3 to 23 months) were shown in favor of the intervention group regarding balance (SMD = 0.512, 95% CI [0.240, 0.785], *p* < 0.001, I^2^ = 87%), gait (SMD = 0.614, 95% CI [0.301, 0.926], *p* < 0.001, I^2^ = 75%), and general motor function (SMD = 0.922, 95% CI [0.559, 1.285], *p* < 0.001, I^2^ = 87%). Heterogeneity among studies was high for all three outcomes, apparently reflecting diversity with regard to patient characteristics, type, and duration of intervention, as well as the method of outcome assessment. The certainty of evidence was consequently judged as ‘’low’’ to ‘’moderate,’’ according to the GRADE system. Subgroup analyses revealed that balance can sustainably improve mostly through multimodal rather than targeted balance-oriented exercise but also through dual-task exercise, tai chi, and Pilates. Gait showed improvement at follow-up mainly through multimodal exercise, aerobic exercise, dual-task exercise, and Pilates, with benefits confined to early- and mid-stage disease. Sustained UPDRS-III improvement could be achieved through multimodal exercise, which showed a large overall effect but also through aerobic, resistance, and dual-task training, tai chi and qigong. Conclusions: Exercise interventions can improve balance and gait, as well as preserve the overall motor function in patients with PD, also in the mid- and long-term post-intervention periods.

## 1. Introduction

Parkinson’s disease (PD) is a chronic, progressive neurodegenerative disorder of the central nervous system [1,2,3,4]. It is associated with the degeneration of dopaminergic neurons in the substantia nigra, leading to dopamine deficiency and basal ganglia dysfunction [3,5,6].

PD is recognized as the most common neurodegenerative disorder after Alzheimer’s disease [5,7]. The disease is characterized by a combination of motor symptoms (rest tremor, bradykinesia, muscular rigidity, and postural and gait impairment) and non-motor symptoms (cognitive, psychiatric, autonomic, and sensory disturbances, sleep disorders, pain, and fatigue) [4,5]. PD mainly affects older adults [7], with incidence ranging from 10 to 18 per 100,000 person-years and the male-to-female ratio being approximately 3:2 [5], the symptoms having a significant impact on the patient’s independence and quality of life.

Balance and gait are complex, multifactorial functions [8] and represent two of the most common impairments in PD, with their progressive decline contributing significantly to disability and mobility loss [9]. Balance is defined as the individual’s ability to maintain the line of gravity within the base of support. This function involves both static balance, which refers to sustaining postural stability and orientation when the body is at rest, and dynamic balance, which involves maintaining control during movement or when transitioning between postures [10,11].

Gait, on the other hand, is a highly coordinated motor process involving the rhythmic and alternating movement of the limbs and trunk to facilitate the forward progression of the body’s center of mass from one location to another [11,12].

There is currently no definitive cure for PD. Management focuses on controlling the symptoms and improving patient functionality [13]. The available medication treatment provides short-term benefits, but long-term use can lead to complications such as motor fluctuations and dyskinesias [5]. Given these limitations, non-pharmacological approaches comprising multiple disciplines, such as physiotherapy, occupational therapy, and speech therapy combined with medication, aim to mitigate symptoms and improve motor functions. Physiotherapy in particular can improve balance and gait, significantly contributing to reducing the risk of falls and enhancing the individual’s independence [14]. Recent studies indicate that a wide variety of physiotherapy interventions substantially improve motor symptoms in PD [15,16,17,18]. Regarding exercise intensity, benefits appear at approximately 60 MET-minutes/week and peak around 1300 MET-minutes/week [16]. Optimal outcomes are achieved with individualized programs of moderate-to-high intensity, 2–3 times/week for 30–60 min [17], also positively impacting quality of life and functional mobility [15,17,18].

Since most studies report only on immediate post-intervention effects of physiotherapy treatments in PD, it is not clear whether and to what extent frequently observed positive effects persist over time. Previously, Mak et al. (2017) [1] have highlighted the positive long-term effects of exercise and physical therapy in PD. Also, Shen et al. (2016) [14] have provided a positive results’ meta-analysis of long-term effects of exercise training on postural stability and fall rate in PD. However, the study considered re-evaluation intervals for long-term effects as short as 4 weeks and did not differentiate between exercise types. Furthermore, the long-term effects on the score of the Unified Parkinson’s Disease Rating Scale (UPDRS), the most commonly used scale for following the longitudinal course of PD, have been understudied. In this context, the aim of the present study was to provide an updated systematic review and meta-analysis summarizing current evidence of mid- and long-term impact of various physiotherapeutic interventions (lasting at least 3 months post-intervention) on dynamic balance and gait, as well as on general motor function in patients with PD. The duration of 3 months is an arbitrary cutoff, which corresponds to a pragmatic trial design and which has also previously been used by others to define durable physiotherapy effects in PD [1]. We hypothesized that these effects might be moderated by factors such as modality of physiotherapeutic treatment, stage of disease, duration and frequency of physiotherapeutic intervention, and duration of post-exercise follow-up. Different interventions might share common mechanisms of action, like, for example, the secretion of exerkines, the induction of immunomodulation, or CNS neuroplasticity, or they can act through diverse mechanisms as well (e.g., aerobic exercise prominently enhances cardiorespiratory fitness and improves metabolic variables, whereas resistance exercise mainly promotes muscle strength and mass). Thus, we intended to further look into which exercise regimens could be associated with carry-over motor benefits lasting at least 3 months after the end of the interventions and to also explore the influence of other above-mentioned factors.

## 2. Methods

A systematic review with meta-analyses was conducted adhering to the 2020 PRISMA (Preferred Reporting Items for Systematic Reviews and Meta-Analyses) guidelines [19], The review protocol was registered in the Open Science Framework (OSF registries identifier: EDYQA).

### 2.1. Data Sources and Search Strategies

A systematic search was conducted between March and November 2025 across the PubMed, Cochrane Library, and Scopus databases to identify controlled clinical trials published from 2000 to 2025, including assessments of various physiotherapy interventions’ effects on balance, gait, and general motor function in PD lasting ≥3 months after the completion of the exercise program.

The articles reviewed were written in English. The time filter was limited to the last 25 years and screening was conducted manually by two researchers (M.H.) and (I.S). Keywords used to identify the articles were: “Parkinson’s disease”, “balance”, “postural control”, “gait”, ‘’motor function’’, ‘’physiotherapy’’, ‘’physical therapy’’, ‘’exercise’’, “dual task training”, “divided attention training”, “virtual reality training”, “exergames”, “resistance training”, “strength exercises”, “aerobic exercise”, “endurance training”, “robotics”, “robot assisted intervention”, “technology”, “wearable”, “cues”, “cueing strategies”, “auditory cues”, “visual cues”, “sensory cues”, ‘’vestibular rehabilitation’’, “tai chi”, “Qigong”, “yoga”, “dance therapy”, “ballroom”, “Pilates”, “music therapy” and ‘’long-term effects’’, and they were combined by means of the Boolean operators AND and OR in the different searches.

An appendix with the complete search strategy is included in the Supplementary Files (File S1).

### 2.2. Eligibility Criteria

#### 2.2.1. Inclusion Criteria

This review considered only studies published in English between 2000 and 2025. The studies were designed as prospective clinical intervention studies with a control group and assessed long-term outcomes of 12 weeks (3 months) or longer after the end of the intervention program. The selection of studies was done according to the PICO (Population, Intervention, Comparison, Outcome(s)) framework, as follows: Population: patients with Parkinson’s disease diagnosed using standard criteria, such as the UK PDS Brain Bank criteria; Intervention: any type of physical intervention, such as aerobic exercise, resistance training, balance and gait training, dual-task training, training with cueing strategies, robot-assisted training, virtual reality-assisted training, tai chi, qigong, Pilates, yoga, dance therapy, and music therapy; Comparison: standard care or other types of intervention used as control; Outcomes: balance capacity, gait parameters, general motor function, frequency of falls, and occurrence of freezing episodes. All studies evaluated balance and/or gait parameters. Although we retrieved and recorded the results of all available studies fulfilling the above criteria, we chose to focus on objectively measured outcomes of dynamic balance, gait capacity, and general motor function obtained through commonly used clinical assessment tools for the meta-analysis to ensure objectivity and clinical applicability of the findings. Therefore, the outcomes derived from self-administered patient questionnaires, such as calendars registering the number of falls or FOG (freezing of gait) questionnaires did not enter the meta-analysis but were recorded and described in a narrative manner to further inform the results of this review. Balance and gait outcomes obtained through electronic gait evaluation systems such as the GAITRite (e.g., gait cadence, gait speed, and other spatiotemporal measures) also did not enter the meta-analysis but were recorded and described narratively. Although digital analysis instruments could provide more sensitive and unbiased assessments compared to conventional tools, the use of such equipment is not available in all clinical facilities and community settings. We further set an intermediate PEDro score of ≥ 5 as a criterion of adequate methodological quality of the studies to be included in the meta-analysis. This threshold was selected to ensure the inclusion of an adequate number of studies for statistical analysis that are at least of fair methodological quality.

#### 2.2.2. Exclusion Criteria

Studies with an irrelevant subject or without a relevant outcome of interest (balance, gait, and motor function), studies without an intervention or with an intervention but without a control group, and studies without the evaluation of long-term outcomes after the completion of the physical therapy program or with post-intervention assessments earlier than 12 weeks (3 months) after the completion of the program were excluded. Duplicate studies, conference abstracts, studies published in a foreign language (other than English), on-going trials, and case studies, as well as studies with insufficient information, were also eliminated from this systematic review. Other systematic reviews and meta-analyses on the subject were excluded from the analysis of the results but were considered in the discussion for comparative reasons.

For the meta-analysis, studies were excluded if they had a PEDro score below 5, if they lacked an appropriate control group, if they did not include a selected outcome of interest, or if they had overlapping samples with other more recent included studies.

The studies were screened and selected by two independent authors. Any discrepancies were resolved by a third reviewer.

### 2.3. Data Extraction

Two independent authors performed the data extraction using a standardized extraction form. Relevant information from eligible studies were extracted including: study characteristics (author, year, sample size, participants’ mean age, sex ratio, and clinical characteristics); intervention details (exercise type, intensity, frequency, and duration); comparator characteristics; outcomes assessed (dynamic and static balance parameters, functional and spatiotemporal gait parameters, general motor function score, frequency of falls, and occurrence of freezing episodes), duration of follow-up period, and the main findings. Where data were incomplete or unclear, attempts were made to contact study authors. Any disagreements in data extraction were resolved through discussion with a third reviewer.

### 2.4. Quality Assessment of the Studies

The methodological quality of the studies was assessed using the PEDro (Physiotherapy Evidence Database) scale, which is based on 11 criteria, 10 of which are scored as follows: (i) eligibility criteria specified (not scored), (ii) random allocation (1 point), (iii) concealed allocation (1 point), (iv) groups similar at baseline (1 point), (v) subject blinding (1 point), (vi) therapist blinding (1 point), (vii) assessor blinding (1 point), (viii) less than 15% dropouts (1 point), (ix) intention-to-treat analysis (1 point), (x) between-group statistical comparisons (1 point), (xi) point measures and variability data (1 point). Scores between 0 and 3 indicate low methodological quality, 4 to 5 indicate moderate methodological quality, 6 to 8 indicate good methodological quality, and 9 to 10 are considered excellent.

### 2.5. Assessment of the Quality of Evidence

The GRADE (Grading of Recommendations, Assessment, Development, and Evaluation) system was used to assess the quality of the evidence from research findings. Quality was defined as the level of confidence in the assessment of the outcome to support a recommendation, considering factors such as risk of bias in studies, inconsistency, imprecision, publication bias, indirectness, and other factors that may affect the reliability of the evidence. The final quality of evidence is rated as high, moderate, low, or very low.

### 2.6. Data Synthesis and Analysis

A meta-analysis of studies based on each of the outcome parameters of interest, namely balance, gait ability, and general motor function was undertaken using the Comprehensive Meta-Analysis Software (CMA V2; Biostat, Inc, Englewood, NJ, USA). Outcome measures entering the meta-analyses included the TUG (timed up and go) test, the BBS (Berg balance scale), the Mini-BESTest, and the Tinetti test for dynamic balance, the 6MWT, the 2MWT, DGI, GABS, Tinetti walking and Hauser ambulation index for gait ability, and the UPDRS-III scores for the overall motor function (or the MDS-UPDRS-III, which corresponds to UPDRS-III, since MDS-UPDRS is a revision of the original UPDRS). In case of studies reporting on both BBS or Mini-BESTest and the TUG test for balance, the BBS or Mini-BESTest was preferred to TUG for meta-analysis as more comprehensive. We focused on the analyses of change scores from the baseline to the longest follow-up assessment performed. The effect sizes were calculated as standardized mean difference (SMD) with a 95% confidence interval to account for different measurement scales used across studies. Where not directly available, reported means and standard deviations from pre- and post-intervention measurements were used to calculate change scores per group in each study for subsequent meta-analysis. Standard deviation of mean change was estimated according to Cochrane guidelines. To handle multi-arm studies, we used the approach of splitting the control group to avoid a unit-of-analysis error (“double counting”). Pooled effect sizes were deemed statistically significant at *p* < 0.05. We classified effect sizes based on Cohen, according to which the SMD values of <0.2 are considered trivial, the values of 0.2 to 0.5 are considered small, the values of 0.5 to 0.8 are considered medium, and the values of >0.8 are considered large. We used the I^2^ values to determine the level of heterogeneity between studies (the values of <25% indicate low, the values of 50–75% indicate moderate, and the values of >75% indicate high heterogeneity). In case of low heterogeneity, the fixed-effects model was used. When significant heterogeneity was present (I^2^ values > 50%), the random-effects model was applied instead. The sensitivity and moderator analyses were conducted to explore the sources of heterogeneity.

Publication bias was tested through visual inspection of funnel plot asymmetry and Egger’s test; the adjustment for publication bias was made via the trim and fill method (the MetricGate tool).

Studies or part of study results that did not enter a meta-analysis are presented in a narrative manner.

## 3. Results

### 3.1. Screening Process

**Figure 1:**
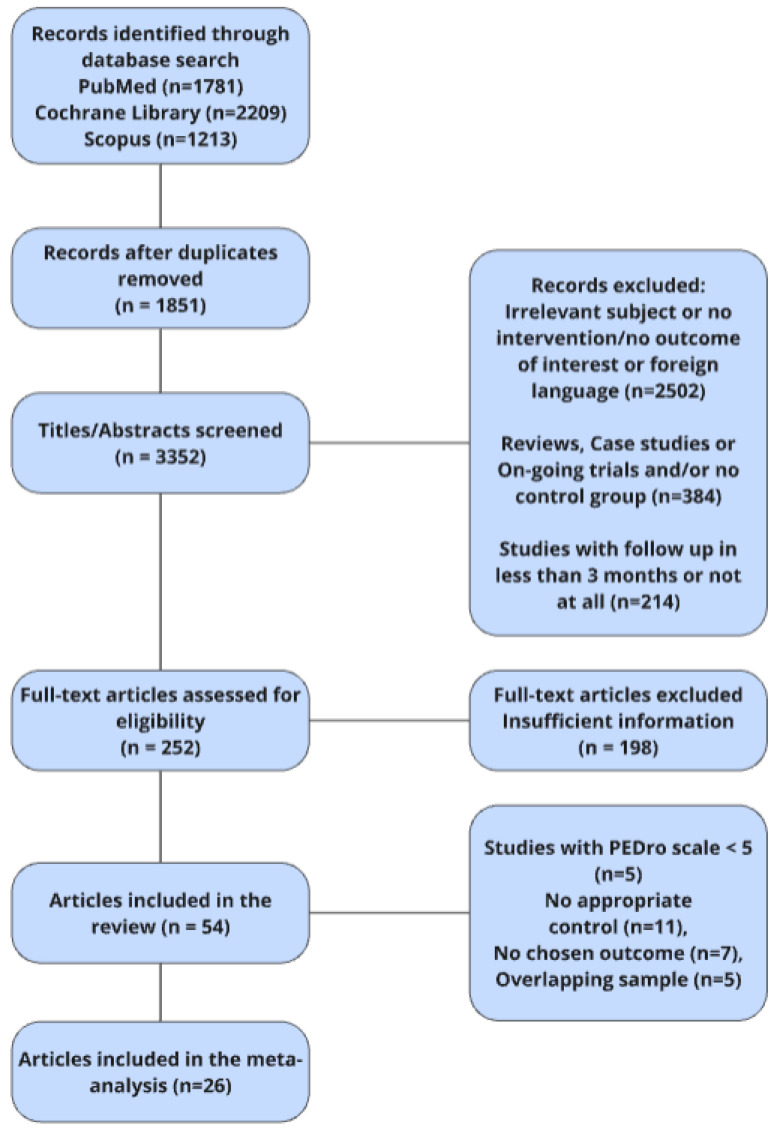
Flowchart of the study selection.

The search yielded 5203 citations after thorough research using the previously described defined keywords. Τaking into account the inclusion and exclusion criteria, 54 studies were identified and systematically evaluated, 26 of which were included in the meta-analyses. The number of subjects from all studies included in the meta-analyses who underwent ≥3 months follow-up assessment reached a total of 1261 participants in the experimental groups and 989 participants in the control groups. The selection process is demonstrated in the flowchart in Figure 1.

Table 1 below summarizes characteristics and main findings of studies included in the meta-analysis. A more detailed table containing all review studies can be found in Supplementary Files (Table S1). The studies were categorized according to intervention type as follows: aerobic training, resistance training, balance and gait training, dual-task exercises, cueing strategy interventions, multimodal physical therapy (either regular or intensive in the form of a rehabilitation program), virtual reality (VR) interventions, robot-assisted training, dance therapy, yoga, tai chi/qigong, Pilates, music therapy, and LSVT BIG for amplitude of movement. Follow-up periods of identified and included studies extended from 3 to 23 months after the completion of each intervention. Studies included in the table but not used in the meta-analyses had either a PEDro score of <5, an unsuitable control group (e.g., control was another distinct active treatment or a variation in the same treatment or the study followed a cross-over design in which the control group converts to the experimental group prior to the follow-up), or a not selected outcome measure for the meta-analysis, or it could possibly show the repetition of the sample with another included study.

### 3.2. Quality Assessments

#### 3.2.1. Evaluation of the Methodological Quality of Studies

The methodological quality of the included studies was assessed using the reliable and valid Physiotherapy Evidence Database Scale (PEDro). The total score a study can receive scores ranging from 0 to 10, with higher scores indicating superior methodological quality. Specifically, scores from 0 to 3 denote low quality, 4 to 5 indicate moderate quality, 6 to 8 indicate good quality, and 9 to 10 indicate excellent quality. Studies scoring 6 or above are generally considered to have high methodological rigor.

Internal quality was evaluated based on PEDro criteria items 2, 3, and 5–9. Each study received a score of 0 or 1 per criterion, depending on whether the requirement was met.

As noted above, the quality of the studies was assessed using the PEDro scale, with scores being presented in Table 2. Fifteen studies demonstrated moderate methodological quality [20,35,37,45,46,47,48,49,50,51,52,53,54,55,56], while 39 studies were classified as methodologically good [13,21,22,23,24,25,26,27,28,29,30,31,32,33,34,36,38,39,40,41,42,43,44,57,58,59,60,61,62,63,64,65,66,67,68,69,70,71,72].

Internal validity was assessed likewise, using the PEDro scale. Twelve studies were identified as having low internal validity [20,35,45,46,47,48,51,52,53,54,55,56], and 29 studies demonstrated moderate internal validity [13,21,24,26,29,31,32,36,37,38,40,42,43,44,49,50,57,58,59,60,61,64,66,67,68,69,70,71,72], while 13 studies were rated as having good internal validity [22,23,25,27,28,30,33,34,39,41,62,63,65].

#### 3.2.2. Evaluation of the Quality of Evidence

The assessment of the quality of evidence through the GRADE system revealed that for all three outcomes (balance, gait, and general motor function), there is ‘’low’’ to ‘’moderate’’ certainty of the evidence presented. All studies were RCTs, so the evidence level started at high. The level of evidence was downgraded by two levels due to very serious inconsistency (very high heterogeneity between studies) for the outcomes of balance and general motor function and by one level due to serious inconsistency regarding the outcome of gait (high heterogeneity between studies). Further, the level of evidence was downgraded by one level due to suspected publication bias for the outcomes of gait and general motor function. The level of evidence was upgraded by one level for the outcome of general motor function due to large effect size. We did not identify other major concerns, in particular other sources of bias, since studies importantly ensured random allocation of subjects to treatment groups and assessor blinding to treatment. Furthermore, inconsistency was reduced when intervention categories were analyzed separately, and positive publication bias results might reflect high heterogeneity rather than actual small study effects.

### 3.3. Main Findings

#### 3.3.1. Balance

**Figure 2:**
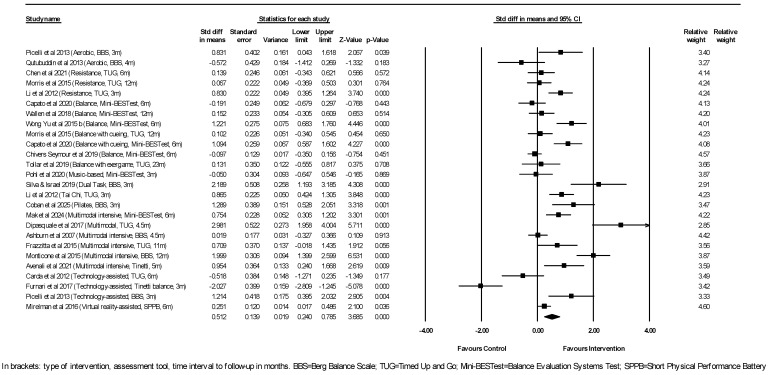
Meta-analysis of studies investigating the sustained effect of physiotherapy interventions on dynamic balance measures in PD patients. Picelli et al., 2013 [21]; Qutubuddin et al., 2013 [20]; Chen et al., 2021 [22]; Morris et al., 2015 [23]; Li et al., 2012 [24]; Capato et al., 2020 [25]; Wallen et al., 2018 [26]; Wong-Yu et al., 2015(b) [27]; Chivers Seymor et al., 2019 [30]; Tollar et al., 2019 [29]; Pohl et al., 2020 [36]; Silva & Israel, 2019 [32]; Coban et al., 2025 [38]; Mak et al., 2024 [13]; Dipasquale et al., 2017 [40]; Ashburn et al., 2007 [39]; Frazzitta et al., 2015 [42]; Monticone et al., 2015 [44]; Avenali et al., 2021 [43]; Carda et al., 2012 [34]; Furnari et al., 2017 [35]; Mirelman et al., 2016 [33].

Regarding balance, 22 studies reporting on dynamic balance outcome measures entered a meta-analysis that included 26 comparisons between experimental interventions and their controls, with a total of 1096 participants in the experimental groups and 831 participants in the control groups (Figure 2).

Significant differences were found in the outcome measure of balance, as assessed by either the BBS, Mini-BESTest, TUG, Tinetti, or SPPB tools, in favor of the intervention group in 13 out of the 26 comparisons. As shown by the random-effects model, physiotherapy interventions were associated with an overall moderately better outcome of dynamic balance ≥ 3 months after cessation of the physiotherapy program compared to the control (pooled SMD = 0.512, 95% CI [0.240, 0.785], *p* < 0.001). Substantial study heterogeneity was indicated by an I^2^ statistic of 87%. Egger’s test *p*-value of 0.58 indicated the absence of publication bias (funnel plot can be found under Supplementary Figure S1). Subsequent subgroup analysis by intervention type to explore this expected main source of heterogeneity revealed that positive effect loaded primarily on multimodal exercise (*N* = 6, SMD = 1.172, 95% CI [0.413, 1.932], *p* = 0.002). A sensitivity analysis, performed by removing the outlier study of Dipasquale et al. [40], further differing from the rest of this subgroup’s studies in the frequency of weekly sessions (x2/week vs. ≥6/week in the other studies), still showed a significant large effect of multi-component exercise on the balance in PD (*N* = 5, SMD = 0.867, 95% CI [0.183, 1.552], *p* = 0.013). Dual-task exercises, tai chi, and Pilates also conferred large positive effects on balance, although these findings were each based on single studies [24,32,38]. Aerobic exercise showed no significant sustained overall effect on dynamic balance (*N* = 2, SMD = 0.138, 95% CI [−1.236, 1.511], *p* = 0.844). Resistance training also did not achieve an overall significant effect on balance (*N* = 3, SMD = 0.349, 95% CI [−0.138, 0.837], *p* = 0.160). Balance training with or without cueing did not reach a statistically significant effect either (*N* = 7, SMD = 0.329, 95% CI [−0.076, 0.734], *p* = 0.11). Robot-assisted exercise did not show a significant effect on balance compared to conventional exercises (*N* = 3, SMD = −0.447, 95% CI [−2.243, 1.349], *p* = 0.625). VR-assisted exercises on the other hand, did provide a small benefit compared to conventional exercises, based on one study [33].

Subgroup analysis by Hoehn & Yahr (including only stage I-III vs. also including stage IV patients with marked balance and gait difficulties) showed that the interventions either confined to early and mid-stage patients (*N* = 16, SMD = 0.445, 95% CI [0.079, 0.811], *p* = 0.017) or extending to more advanced disease stages (*N* = 10, SMD = 0.619, 95% CI [0.174, 1.064], *p* = 0.006) were in both cases associated with a positive outcome. Subgroup analysis by duration of the intervention program (<3 months vs. ≥3 months) showed that longer-duration programs yielded more prominent effects (*N* = 9, SMD = 0.793, 95% CI [0.329, 1.257], *p* = 0.001) than the shorter-duration ones (*N* = 17, SMD = 0.359, 95% CI [0.008, 0.710], *p* = 0.045). Time interval to follow-up assessment (<6 months vs. ≥6 months) did not influence outcomes. Both follow-ups 3 to 5 months after the cessation of the intervention programs (*N* = 12, SMD = 0.673, 95% CI [0.113, 1.234], *p* = 0.019), as well as longer follow-ups after 6 months and up to 23 months (*N* = 14, SMD = 0.405, 95% CI [0.112, 0.697], *p* = 0.007) showed positive effects.

#### 3.3.2. Gait

**Figure 3:**
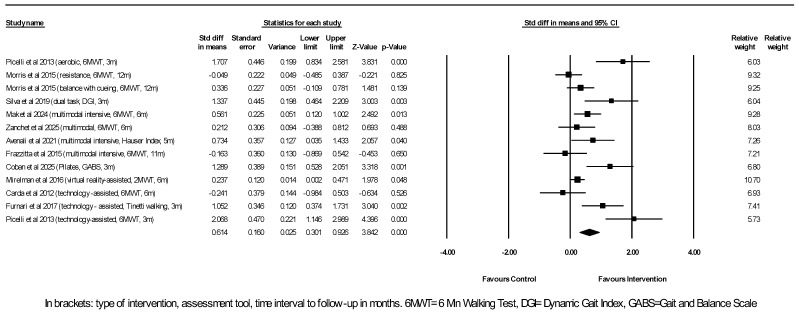
Meta-analysis of studies investigating the sustained effect of physiotherapy interventions on gait capacity measures in PD patients. Picelli et al., 2013 [21]; Morris et al., 2015 [23]; Silva & Israel, 2019 [32]; Coban et al., 2025 [38]; Mak et al., 2024 [13]; Frazzitta et al., 2015 [42]; Avenali et al., 2021 [43]; Carda et al., 2012 [34]; Furnari et al., 2017 [35]; Mirelman et al., 2016 [33]; Zanchet et al., 2025 [41].

Regarding gait capacity, 11 studies reporting on gait capacity outcome measures entered a meta-analysis that included 13 comparisons between experimental interventions and their controls, with a total of 480 participants in the experimental groups and 366 participants in the control groups (Figure 3).

Significant differences were found for the outcome measure of gait capacity, as assessed by either the 6MWT, DGI, Hauser index, GABS, 2MWT, or Tinetti walking assessment tools in favor of the intervention group in eight out of the 13 comparisons. As shown by the random-effects model, the physiotherapy interventions were associated with an overall moderately better outcome of gait capacity ≥ 3 months after the cessation of the physiotherapy program compared to the control (pooled SMD = 0.614, 95% CI [0.301, 0.926], *p* < 0.001). The I^2^ statistic of 75% showed substantial heterogeneity between studies. Egger’s test *p*-value of 0.04 indicated possible publication bias or could reflect between-studies heterogeneity (funnel plot can be found under Supplementary Figure S2). The correction with the trim and fill method showed a still highly significant adjusted effect estimate. Subgroup analysis by intervention type focusing on only multimodal exercise interventions showed a significant small positive effect (*N* = 4, SMD = 0.388, 95% CI [0.099, 0.677], *p* = 0.009). Aerobic exercise, dual-task exercise, and Pilates conferred large positive effects on gait capacity, though each was derived from single reports [21,32,38]. Regarding robot-assisted interventions, although two studies [21,35] showed significant large positive effects on gait capacity 3 months post-exercise, the overall effect was not significant due to a third negative study conducting its follow-up assessment 6 months post-exercise [34] (*N* = 3, SMD = 0.937, 95% CI [−0.300, 2.175], *p* = 0.138). As with the balance outcome, VR-assisted exercise provided a small benefit compared to conventional exercise based on one study [33].

The subgroup analysis by Hoehn & Yahr stages (including only stage I–III vs. also including stage IV patients with marked balance and gait difficulties) showed that the interventions confined to early- and mid-stage patients yielded an overall significant modest positive effect on gait (*N* = 10, SMD = 0.680, 95% CI [0.295, 1.065], *p* = 0.001), whereas the interventions extending to more advanced disease stages did not (*N* = 3, SMD = 0.435, 95% CI [−0.193, 1.063], *p* = 0.175).

The subgroup analysis by the duration of the intervention program (<3 months vs. ≥3 months) showed that the longer-duration programs yielded positive effects (*N* = 3, SMD = 0.636, 95% CI [0.110, 1.162], *p* = 0.018) of similar magnitude to the shorter-duration ones (*N* = 10, SMD = 0.615, 95% CI [0.227, 1.002], *p* = 0.002). On the other hand, time interval to follow-up assessment (<6 months vs. ≥6 months) did influence outcome. The follow-up 3 to 5 months after the cessation of the intervention program showed an overall large positive effect (*N* = 6, SMD = 1.276, 95% CI [0.956, 1.597], *p*< 0.001), whereas longer follow-up after 6 months and up to 12 months still showed an overall positive effect but of smaller magnitude (*N* = 7, SMD = 0.210, 95% CI [0.052, 0.368], *p* = 0.009).

#### 3.3.3. General Motor Function

**Figure 4:**
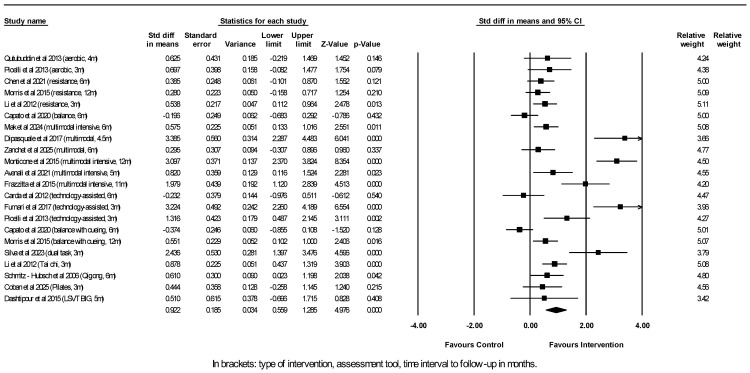
Meta-analysis of studies investigating the sustained effect of physiotherapy interventions on UPDRS-III motor score in PD patients. Picelli et al., 2013 [21]; Qutubuddin et al., 2013 [20]; Chen et al., 2021 [22]; Morris et al., 2015 [23]; Li et al., 2012 [24]; Capato et al., 2020 [25]; Coban et al., 2025 [38]; Mak et al., 2024 [13]; Dipasquale et al., 2017 [40]; Frazzitta et al., 2015 [42]; Monticone et al., 2015 [44]; Avenali et al., 2021 [43]; Carda et al., 2012 [34]; Furnari et al., 2017 [35]; Zanchet et al., 2025 [41]; Silva et al., 2023 [31]; Schmitz-Hubsch et al., 2006 [37]; Dashtipour et al., 2015 [45].

Regarding general motor function as assessed by either the UPDRS-III or the MDS-UPDRS-III, which correspond to one another, 18 studies reporting on this outcome in the long run entered the meta-analysis that included 22 comparisons between experimental interventions and their controls, with a total of 716 participants in the experimental groups and 449 participants in the control groups (Figure 4).

Significant differences were found in the outcome measure of general motor function in favor of the intervention group (improvement or reduced deterioration over time compared to control) in 12 out of the 22 comparisons. As shown by the random-effects model, physiotherapy interventions were associated with an overall considerably better outcome regarding UPDRS-III score ≥ 3 months after the cessation of the physiotherapy program compared to control (pooled SMD = 0.922, 95% CI [0.559, 1.285], *p* < 0.001). The I^2^ statistic of 87% showed substantial heterogeneity between studies. Egger’s test *p*-value of 0.005 indicated possible publication bias or could reflect between-studies heterogeneity (funnel plot can be found under Supplementary Figure S3). The correction with the trim and fill method showed a still highly significant adjusted effect estimate. A further subgroup analysis by intervention type showed that multimodal exercise exerted a large significant positive effect on the UPDRS-III scores at follow-up (*N* = 6, SMD = 1.641, 95% CI [0.655, 2.628], *p* = 0.001), loading primarily on intensive ≥5 sessions/week rehabilitation programs (*N* = 4 studies, SMD = 1.597, 95% CI [0.422, 2.772], *p* = 0.008). On the other hand, regular multimodal interventions with a frequency of treatment of x2/week did not reach a statistically significant effect (*N* = 2, SMD = 1.804, 95% CI [−1.224, 4.832], *p* = 0.243). Moderate significant positive effects on UPDRS-III also derived from aerobic exercise (*N* = 2, SMD = 0.664, 95% CI [0.092, 1.237], *p* = 0.023) and resistance training (*N* = 3, SMD = 0.405, 95% CI [0.146, 0.663], *p* = 0.002), while dual-task training, tai chi and qigong also showed positive effects based on single reports [24,31,37]. Isolated balance training had no effect on UDRS-III scores at follow-up (*N* = 3, SMD = 0.000, 95% CI [−0.569, 0.569], *p* = 0.999). Robot-assisted training did not lead to overall significantly better UPDRS-III sores at follow-up compared to conventional therapies (*N* = 3, SMD = 1.416, 95% CI [−0.502, 3.334], *p* = 0.148).

Subgroup analysis by Hoehn & Yahr (including only stage I-III vs. also including stage IV patients with marked balance and gait difficulties) showed that interventions either confined to early and mid-stage patients (*N* = 14, SMD = 0.844, 95% CI [0.353, 1.335], *p* = 0.001) or extending to more advanced disease stages (*N* = 8, SMD = 1.060, 95% CI [0.501, 1.620], *p* < 0.001) were in both cases associated with a positive outcome.

The subgroup analysis by duration of the intervention program (<3 months vs. ≥3 months) showed that longer-duration programs yielded positive effects on the UPDRS-III score (*N* = 8, SMD = 0.747, 95% CI [0.358, 1.137], *p* < 0.001), as did the shorter-duration ones (*N* = 14, SMD = 1.013, 95% CI [0.440, 1.586], *p* = 0.001).

Furthermore, time interval to follow-up assessment (<6 months vs. ≥6 months) appeared to influence this outcome. Follow-up 3 to 5 months after the cessation of the intervention program showed an overall large positive effect on the UPDRS-III score (*N* = 11, SMD = 1.284, 95% CI [0.758, 1.810], *p* < 0.001), whereas longer follow-up after 6 months and up to 12 months still showed an overall positive effect but of smaller magnitude (*N* = 11, SMD = 0.597, 95% CI [0.118, 1.076], *p* = 0.015).

Since possible anti-PD medication adjustments during follow-up are an important caveat when it comes to interpreting the UPDRS-III change scores in PD, we reviewed the studies with regard to this aspect. Six out of the 18 studies on change in the UPDRS scores fail to report on PD medications. The studies by Morris et al. (2015) [23] and Monticone et al. (2015) [44] record and compare anti-PD medication between the intervention and control groups only at baseline but omit to investigate drug modifications at follow-up. However, most of the studies take into account and control for alterations in medication use during follow-up, like Li et al. (2012) [24], who closely monitored the dose and frequency of medication during their trial conducted via the Medication Change Questionnaire (MCQ), while Capato et al. (2020) [25], Mak et al. (2024) [13], Zanchet et al. (2025) [41], Frazzitta et al. (2015) [42] and recorded the LEDD (levodopa equivalent daily dose) at different time points during follow-up. Furthermore, Carda et al. (2012) [34] and Furnari et al. (2017) [35] record medication at baseline and report that none of the patients modified his or her drug treatment during the study. Dashtipour et al. (2015) [45], without detailing medications, state that there were no changes in medications related to PD during the study. Coban et al. (2025) [38] stated that they excluded patients that had to change their medication or dosage during the treatment phase without providing detailed descriptions of medications. Schmitz-Hübsch et al. (2006) [37] report that 26% in the treatment and 40% in the control group increased the dose of anti-PD medication at 6-month follow up but that such increases more often resulted in the deterioration of motor scores and were thus unlikely to have contributed to the observed benefits of qigong therapy. Finally, Frazzitta et al. (2015) [42] actually used LEDD as a secondary outcome in their study and demonstrated that the intervention group was able to retain a lower dose of anti-PD medication at follow-up compared to the control group.

#### 3.3.4. Additional Findings of Studies and Outcomes (Spatiotemporal Gait Parameters, Falls, and Freezing of Gait) Not Included in the Meta-Analyses

The studies included in this review but not assessed through meta-analyses provide further insights into long-term effects of physiotherapy interventions on outcomes associated with balance and gait. The main findings of studies are listed in the last column of Table 1. Aerobic training can improve gait speed [46], functional mobility, FOG [47], and stride length [21], as well as various gait parameters such as speed, step length, single-leg support, and swing phase [48], at 3 months post-exercise.

Balance training alone did not provide a retention effect in terms of dynamic balance outcomes, as shown in the meta-analysis. In support of this, another study by Rennie et al. demonstrated short-term improvements in various spatiotemporal measures achieved through the HiBalance program that were not maintained at 6- and 12-month follow-ups [50]. Furthermore, a high-intensity and high-frequency 3-week-long exergaming program comprising gait, coordination, posture, and balance exercises yielded detraining effects in the experimental group lasting up to 18 months [29] or even 36 months [60] regarding the performance on the TUG test. However, these effects gradually further worsened and ultimately did not diverge from the control at the end of the follow-up. The inclusion of an experimental plus maintenance group in these studies helped prove that, for lasting exercise-induce neuroprotective and restorative effects to occur, participation in a long-term exercise maintenance program is required. PDSAFE, a balance and fall avoidance strategy program was not effective in reducing falls in PD overall [30]. Rhythmic auditory cueing might be more beneficial than conventional balance training, as shown in the study by Capato et al. [25]. Physiotherapy plus cognitive training is not superior for the outcome balance compared to the isolated motor physiotherapy, although both led to improvement in balance after 3 months of the follow-up [59].

Both the tai chi and resistance training groups showed a reduction in the number of falls 3 months after completion of the training in the study by Li et al. [24], with greater benefit maintained in the tai chi group. A further study by Gao et al. [68] also showed a reduced number of falls in the tai chi group compared to the control group 6 months after the end of the intervention. Both the study by Li et al. and that by Gao et al. included patients with Hoehn and Yahr stage ≥3, i.e., those with clinically manifest balance problems. Thus, these findings point to tai chi as a promising intervention for the affected patients.

The study by Morris et al. (2015) [23] demonstrated a long-term reduction in the fall rate achieved through both resistance training exercise and movement strategy training. Resistance and balance training seemed to be of similar efficacy in improving functional mobility when compared to one another [58].

Cueing might confer short-term benefits but neither meta-analysis results nor other studies on this type of intervention could prove lasting effects, like in the study by de Icco et al. [53], in which gains were lost at 3 months.

Technology-assisted training, mainly involving the use of exoskeletons, did not confer additional long-term benefits over the conventional physiotherapy methods, as shown in the meta-analysis of objective measures of balance and gait. However, Shen et al. 2015 [65], implementing a 12-week technology-assisted gait and balance training program, demonstrated a reduction in falls, along with a reduction in the postural response latency and an increase in the stride length in the intervention group. Perceived self-reported balance capacity, as well as spatiotemporal parameters such as single-leg support time, might improve more in the intervention group even in the post-exercise phase [64,65].

Regarding yoga, moderate improvements were observed in MDS-UPDRS-III immediately post-intervention and at 3 months, while no changes were seen in TUG in the study by Kwok et al. [67], while in the study of Cheung et al. [66], improvements in mobility were not maintained long-term.

The duration of a physiotherapy treatment might play a role when it comes to the long-term maintenance of balance improvement achieved through exercise, as indicated in the meta-analysis. In line with this finding, the study by Pelosin et al. [51] showed that the 12-week group showed greater improvements in fall frequency compared to the 6-week group after treadmill and VR training at the 6 months follow-up.

The frequency of training is another aspect that might influence outcomes. Nevertheless, multimodal exercise performed two or three times a week yielded comparable long-term positive effects to intensive rehabilitation programs. To further support the notion that a high frequency of training might not be as decisive in improving motor outcomes, Pelosin et al. 2017 showed that high frequency aerobic exercise does not necessarily bring better results than low or intermediate frequency exercise 4 months post-intervention [57].

**Table 2:** PEDro scale scores of included studies.

Author	Year	1	2	3	4	5	6	7	8	9	10	11	Total
Mirelman et al. [33]	2016	1	1	1	1	0	0	1	1	1	1	1	8/10
Pelosin et al. [51]	2022	0	1	0	1	0	0	1	0	0	1	1	5/10
Carda et al. [34]	2012	1	1	1	1	0	0	1	1	1	1	1	8/10
Furnari et al. [35]	2017	0	1	0	1	0	0	1	0	0	1	1	5/10
Picelli et al. [21]	2013	0	1	0	1	0	0	1	1	1	1	1	7/10
Shen & Mak [65]	2015	1	1	1	1	0	0	1	1	1	1	1	8/10
Shen & Mak [64]	2014	1	1	0	1	0	0	1	1	1	1	1	7/10
Da Silva, Iucksch & Israel [31]	2023	1	1	1	1	0	0	1	1	0	1	1	7/10
Geroin et al. [61]	2018	0	1	0	1	0	0	1	1	1	1	1	7/10
Silva & Israel [32]	2019	1	1	1	1	0	0	1	1	0	1	1	7/10
Strouwen et al. [62]	2017	0	1	1	1	0	0	1	1	1	1	1	8/10
Chen et al. [22]	2021	1	1	1	1	0	0	1	1	1	1	1	8/10
Mak et al. [13]	2024	1	1	1	1	0	0	1	0	1	1	1	7/10
Miyai et al. [56]	2002	1	1	0	1	0	0	0	0	0	1	1	4/10
Pelosin et al. [57]	2017	1	1	0	1	0	0	1	1	0	1	1	6/10
Qutubuddin et al. [20]	2013	1	1	0	1	0	0	1	0	0	1	1	5/10
Rawson et al. [46]	2019	1	0	0	1	0	0	1	0	0	1	1	4/10
Wroblewska et al. [47]	2019	1	1	0	1	0	0	0	0	0	1	1	4/10
Stuckenschneider et al. [48]	2015	1	1	0	1	0	0	0	1	0	1	1	5/10
Cheung et al. [66]	2018	1	1	1	1	0	0	1	1	0	1	1	7/10
Gao et al. [68]	2014	1	1	0	1	0	0	1	1	0	1	1	6/10
Li et al. [24]	2012	1	1	0	1	0	0	1	1	1	1	1	7/10
Pohl et al. [36]	2020	1	1	0	1	0	0	1	1	0	1	1	6/10
Schmitz-Hubsch et al. [37]	2006	0	1	0	1	0	0	0	1	1	1	0	5/10
Coban, Kaygisiz & Selcuk [38]	2025	1	1	1	1	0	0	0	1	0	1	1	6/10
Kwok et al. [67]	2019	1	1	1	1	0	0	1	0	1	1	1	7/10
Capato et al. [25]	2020	1	1	1	1	0	0	1	1	1	1	1	8/10
De Icco et al. [53]	2015	1	1	0	1	0	0	0	0	0	1	1	4/10
Murgia et al. [54]	2018	1	1	0	1	0	0	1	0	0	1	1	5/10
Ashburn et al. [39]	2007	1	1	1	1	0	0	1	1	1	1	1	8/10
Avenali et al. [43]	2021	1	1	0	1	0	0	1	1	0	1	1	6/10
Dashtipour et al. [45]	2015	1	1	0	1	0	0	0	1	0	1	1	5/10
Dipasquale et al. [40]	2017	1	1	1	1	0	0	1	0	1	1	1	7/10
Ellis et al. [70]	2005	1	1	1	1	0	0	1	1	0	1	1	7/10
Frazzitta et al. [71]	2012	1	1	0	1	0	0	1	1	0	1	1	6/10
Frazzitta et al. [42]	2015	0	1	1	1	0	0	1	0	0	1	1	6/10
Monticone et al. [44]	2015	1	1	1	1	0	0	0	1	0	1	1	6/10
Morris, Iansek & Kirkwood [63]	2009	1	1	1	1	0	0	1	1	1	1	1	8/10
Palamara et al. [72]	2017	1	1	1	1	0	0	1	1	0	1	1	7/10
Rennie et al. [50]	2021	1	1	0	1	0	0	0	1	1	0	1	5/10
Sparrow et al. [49]	2016	1	1	1	0	0	0	1	0	0	1	1	5/10
Wallen et al. [26]	2018	1	1	0	1	0	0	0	1	1	1	1	6/10
Wong-Yu & Mak [28]	2015(a)	1	1	1	1	0	0	1	1	1	1	1	8/10
Morris et al. [23]	2015	1	1	1	0	0	1	1	1	1	1	1	8/10
Li et al. [69]	2014	0	1	0	1	0	0	1	1	1	1	1	7/10
Terra et al. [59]	2020	1	1	1	1	1	0	0	1	1	1	1	8/10
Wong-Yu & Mak [27]	2015(b)	1	1	1	1	0	0	1	1	1	1	1	8/10
Zanchet et al. [41]	2025	1	1	1	1	0	0	1	1	1	1	1	8/10
Schlenstedt et al. [58]	2015	0	1	0	1	0	0	1	0	1	1	1	6/10
Martin et al. [55]	2015	1	1	0	1	0	0	0	1	0	1	1	5/10
Hortobágyi et al. [60]	2021	1	1	1	0	0	0	1	0	1	1	1	6/10
Tollár et al. [29]	2019	1	1	1	1	0	0	1	0	0	1	1	6/10
Chivers-Seymor et al. [30]	2019	1	1	1	1	0	0	1	1	1	1	1	8/10
Pelosin et al. [52]	2020	0	1	0	1	0	0	1	0	0	1	0	4/10

## 4. Discussion

This systematic review and meta-analysis examined the sustained effects of various physiotherapeutic techniques on balance, gait, and motor function in patients with Parkinson’s disease. Although a number of reviews have previously highlighted the beneficial role of exercise in PD [1,14,73], we rather focused on the retention effects of exercise on diverse motor outcomes. Traditionally, a washout period is applied in clinical trials to test for disease modulatory effects of dopaminergic medication [74]. In line with Mak et al.’s review published in 2017 [1], we evaluated long-term effects of interventions with a minimum of 12 weeks of follow-up after the treatment ended. The majority of studies evaluating the maintenance of beneficial effects in the mid- and long-term demonstrated significant improvements at the evaluated time points (≥3 months after the completion of each intervention program) in balance, gait, and motor performance. However, some studies reported either no significant improvements or a decline in benefits obtained immediately post-intervention as time progressed. A total of 54 studies were included in the review, 26 out of which were incorporated into meta-analyses, which helped resolve conflicting findings between studies and provide cumulative effect estimates of the interventions on outcomes of interest. The follow-up periods extended from 3 to 23 months after the completion of each intervention. Positive cumulative effects at the post-exercise follow-up were shown in favor of the intervention group regarding all three outcomes of balance, gait, and general motor function. These variables were assessed using valid and reliable tools, including time up and go (TUG), 6-min walk test (6MWT), and the Unified Parkinson Disease Rating Scale Part III (UPDRS-III). In particular, the large effect size for the UPDRS-III score placing the mean change in the treatment group approximately one standard deviation apart from the mean change in the control group is indicative of relevant clinical significance, considering that levodopa typically induces an improvement in the MDS-UPDRS Part III (motor) scores by approximately 30% when comparing ‘’on’’ to ‘’off’’ states.

Subgroup analyses revealed that balance can sustainably improve mostly through multimodal rather than targeted, balance-orientated exercise but also through dual-task exercise, tai chi, and Pilates, with longer duration of the intervention programs associated with better outcomes. Gait showed improvement at follow-up mainly through multimodal exercise, aerobic exercise, dual-task exercise, and Pilates, with benefits confined to early- and mid-stage disease. Sustained UPDRS-III improvement could be achieved through multimodal exercise, which showed a large overall effect, but also through aerobic and resistance training, as well as dual-task training, tai chi, and qigong. Regarding gait and UPDRS-III, a longer follow-up period was overall associated with attenuated but still significant effects.

Although the majority of published studies reported positive effects, there are also several studies that did not demonstrate the maintenance of positive effects over time, or in some cases, even a worsening of performance compared to the control after the end of the intervention. Different reasons might account for failure of studies to show positive effects. For instance, in the study by De Icco et al. [53], although the frequency of sessions was higher (five vs. two sessions per week) compared to other studies applying cueing strategies, shorter intervention duration (4 weeks), and/or more advanced stage of PD may have limited the long-term effectiveness of the intervention. Likewise, Morris et al. (2009) [63] reported no improvements in balance-related outcomes and only minimal retention of gains in the 2MWT. These findings may be attributed to the very short intervention period (2 weeks), the limited daily session duration (approximately 37.6 min), and/or the small sample size, possibly rendering the study underpowered to detect a significant effect (totally 28 participants). Furnari et al. (2017) [35] also reported a long-term decline in outcomes, although partial retention of gains was observed. This may be attributed to the absence of home-based practice in contrast to other studies such as those by Shen and Mak (2014, 2015) [64,65], which incorporated additional self-training (20 min daily) and greater stimulation, leading to more consistent results. Three studies that investigated exoskeleton-based interventions over a similar period, each with 12 sessions (three sessions per week) but with varying durations (30, 60, and 45 min) and PD stages (H&Y ≤3, 2–3, and 3) [21,34,35]. Similarly, studies that examined aerobic exercise differed in both protocol design and disease severity of their participants, which may explain the observed declines and discrepancies regarding sustained benefits [46,48,57]. Studies showed limited or diminishing long-term effects, which are likely due to advanced or heterogeneous disease stages or excessive training intensity [57] and short intervention duration without home-based reinforcement. In contrast, studies that demonstrated maintained benefits, such as that by Wroblewska et al., 2019 [47], applied longer intervention durations, moderate intensity, and early disease stages, and they included additional self-practice, promoting adherence and neuroplastic adaptation. Notably, Stuckenschneider et al. (2015) [48], despite involving advanced-stage participants, observed only temporary improvements that were partially sustained over time. Overall, these comparisons suggest that early-stage interventions of moderate intensity, sufficient duration, and home-based exercise are key factors for achieving long-term effectiveness in exercise-based programs for PD.

A main finding of our study is that multimodal programs generally demonstrated considerable long-term benefits in the outcomes of interest. There were exceptions, like the study by Ashburn et al. (2007) [39], which showed a reduction or lack of sustained benefits. In Ashburn et al. (2007) [39], the decline in outcomes, except for falls and functional reach test, may be attributed to the participants’ advanced age, inclusion of later disease stages (H&Y II-IV), the short intervention period (6 weeks), and the extended follow-up duration. Also, it is possible that other individual factors determine the outcome of each study, like the exact components of the multimodal exercise regime. Interestingly, the fact that effect sizes were larger for multimodal training programs compared to several single-modality interventions implies synergistic or additive effects that certainly warrant further investigation. Among intensive multimodal rehabilitation programs, one study with older participants diagnosed with PD-MCI, specifically the one by Avenali et al. (2021) [43], despite a relatively short intervention duration (4 weeks), showed that the presence of a cognitive impairment mild degree does not hinder long-term motor outcome retention.

On the other hand, balance and gait training appeared less consistently effective in promoting long-term functional benefits in PD. As previously mentioned, Capato et al. (2020) [25] reported sustained gait improvements at 6 months only in the group receiving rhythmic auditory stimulation, possibly due to the increased sensory input provided to that group. On the contrary, the studies by Wállen et al. (2018) [26] and Rennie et al. (2021) [50] did not observe maintained gains at 6 and 12 months post-intervention, respectively. Both studies implemented the HiBalance program, unlike others in the same category [26,50]. Despite having large sample sizes, in the participants in early-to-moderate disease stages and in interventions lasting over 10 weeks, long-term retention was not achieved. Older age of participants (inclusion criterion >60 years for the study by Wallen et al., mean age 73 years in the study by Rennie et al.) could possibly have played a role for this lack of effect.

The findings of the present systematic review and meta-analysis align with those of the meta-analysis by Shen, Wong-Yu, and Mak (2016) [14], who examined the effects of exercise-based training on balance, gait, and fall incidence. The positive outcomes observed in our analysis are consistent with their conclusions. Specifically, the effect size of Hedges’ g = 0.419 for improvement of balance and gait performance over the long-term observed in the meta-analysis by Shen et al. is comparable to the effect sizes of 0.512 for balance and 0.614 for gait found in our meta-analyses. Although a meta-analysis on fall rates was not conducted in the current study, a closer examination of the included trials suggests a noticeable reduction in the number of falls among those that evaluated this parameter in the long term. This contrasts with Shen, Wong-Yu, and Mak (2016) [14], who reported no significant short- or long-term effects of interventions on fall rates.

In addition, a narrative review by Mak et al. (2017) [1], which analyzed the effects of both long-term interventions and long-term follow-up, further supports the current findings. The study concluded that such physiotherapeutic approaches lead to improvements in balance, gait, fall prevention, and overall motor performance in individuals with Parkinson’s disease [1]. Interestingly, also in agreement with our results, different exercise modalities can have positive effects on the (MDS)-UPDRS-III score that are roughly comparable to the effects of dopaminergic medication, as stated in another recent review with meta-analyses not applying a duration of follow-up filter [73].

The positive effects of exercise on motor state in PD patients that persist over time could be explained through exercise-induced neuroplasticity. Intriguingly, a recent neuroimaging study showed increased dopamine transporter availability in both the substantia nigra and putamen and an increase in neuromelanin concentration in the substantia nigra in PD patients after 6 months of high-intensity interval training program, suggesting a role for exercise as an effective non-invasive neuromodulatory therapy [75]. Of specific importance in PD, exercise can induce adaptations in the basal ganglia, such as facilitation of the dopamine production pathway, an increase in striatal dopamine (D2) receptors, and a reduction in oxidative stress markers [76,77]. Increased neuroplasticity through exercise might be mediated by enhanced brain-derived neurotrophic factor (BDNF)-tyrosine receptor kinase B (TrkB) signaling [78]. Interestingly, animal models of PD have shown the recovery of motor function through exercise [79]. Demonstrating a true disease-modifying or neuroprotective effect of exercise in patients would require robust clinical trials designs with larger cohorts and substantially longer follow-up periods than those included in the present analysis. The emerging concept of motor reserve provides a plausible interpretation of the observed long-term motor benefits in PD through exercise. Increasing evidence suggests that sustained improvements following exercise-based interventions may reflect the enhancement or preservation of motor reserve, i.e., the buildup of resilience mechanisms such as lower neuroinflammation and greater neuronal substrate, which in turn may actively foster network adaptations to counteract motor decline in PD [80].

A clinical practice guideline issued by the American Physical Therapy Association with the intention to guide physical therapy treatment in PD demonstrated the benefits of various exercise modes in PD after reviewing the literature and emphasized the need for future research to determine optimal modes and dosing of treatment and to determine lasting effects [81]. For lasting improvement in balance, the interventions with a treatment duration of ≥3 months did provide a positive overall effect in our analysis, while those with duration of <3 months did not. Regarding the duration of physiotherapy programs, several studies were able to demonstrate positive effects after long duration intervention protocols and/or maintenance treatment [60,82,83]. These studies assessed patients at long-term, but the intervention also lasted as long. Although highlighting the importance of ongoing physical therapy in the disease, immediate post-exercise effects in PD patients prone to sedentary lifestyle might be symptomatic and in part reflect short-lived improvements in cardiorespiratory fitness, muscle strength, and agility. In their review and based on available evidence at that point, Mak et al. concluded that a minimum of 4 weeks of gait training or 8 weeks of balance training can have positive effects that persist for 3–12 months after treatment completion, while a longer duration of at least 12 weeks of sustained strength training, aerobic training, tai chi, or dance therapy can also produce long-term beneficial effects [1].

In our study, although positive long-term effects on gait performance and the UPDRS-III score were mitigated with longer follow-up durations (>6 months), as shown in the sensitivity analysis, the results remained statistically significant, pointing to the possibility of residual neuroplasticity changes underlying these effects. As shown by Frazzitta et al., a repeat session of a 28-day multidisciplinary intensive rehabilitation treatment at 1-year interval might help preserve or boost the effects observed at 24 months follow-up, leading to the assumption that the intervention might slow down the progression of motor decay in the disease [42].

Our study showed that the timing of intervention earlier in the course of disease might influence the ability to maintain benefits in gait through exercise. This might indeed be the case for potential disease-modifying effects, whereas advanced stages might mainly experience immediate symptomatic benefits. However, not all studies support early interventions [84].

Strengths of our study are: first, the focus on the persistent effects of exercise in PD lasting at least 3 months after the exercise stimulus is withdrawn; second, the utilization of clinician-administered objective outcome measures, with the majority of studies reporting assessor blinding; and third, the implementation of robust meta-analysis including studies of overall adequate quality with low drop-out rates and examining for publication bias to enhance precision and reliability of results. Another advantage of the study is the consideration of multiple outcome measures relevant to balance and gait, even if not selected for the meta-analysis, to approach efficacy of the interventions in various dimensions. This study also has limitations, which should be considered. First, the number of studies investigating long-term outcomes was limited within each intervention category, and many of the studies recruited small sample sizes, reducing the strength of conclusions that can be drawn. Additionally, the methodological heterogeneity across studies (intervention protocols, assessment tools, functional mobility, and the UPDRS motor score assessed either in the “on” or “off” state) might prevent the generalization of the results. Heterogeneity might also arise from the use of the “longest follow-up” (3–23 months). Further, the multimodal exercise category included interventions with variable components in terms of intensity, frequency, and duration. This heterogeneity may limit the ability to identify which specific elements are driving the observed effects. Regarding timing of clinical assessments, these were done either only in the ‘’on’’ state [23,25,35], or the assessment state was not specified in the methods description of the studies. Although we exploratively performed a subanalysis of studies by included Hoehn and Yahr stages, the duration of treatment protocols and length of post-exercise interval to follow-up, as well as the information on the severity of the disease at baseline was not uniformly presented in studies, and the available data were not sufficient to simultaneously control for all potential moderators. Despite UPDRS-III being widely used to assess motor impairment in Parkinson’s disease, it has relevant limitations in capturing disease progression and subtle longitudinal changes. Prior work has shown limited sensitivity of UPDRS-III over time [85]. Therefore, the measures of functional mobility and gait or motor symptom subdomain scores may offer higher sensitivity compared with global clinical scales. Furthermore, not all studies reported on anti-PD medication adjustments during the follow-up, which might have influenced the results, in particular the change in the UPDRS-III motor scores. Nevertheless, based on the studies that did consider the factor of anti-PD medication dose, positive motor outcomes through physiotherapy interventions seem to be independent of drug modifications during the follow-up and might in some cases even allow slower increases in dopaminergic medication dose over time. Whether this could lead to reduced motor complications by sparing levodopa in the long-term remains to be investigated. Regarding the length of the follow-up interval, it is possible that positive effects of studies with short-duration of follow-up could have waned had it been for longer follow-up period. Since most studies did not target a specific age group of PD patients—with only a few exceptions, like the study by Wallen et al. [26], which included patients of >60 years old and reported that the mean age of participants in all studies fell in their sixties up to max mid-seventies, we did not perform a differentiated analysis by age. Moreover, most interventions were applied to cognitively unimpaired subjects. Although Avenali et al. [43] did provide encouraging results, the fact is that few studies have investigated the long-term preservation of cognitive functions in PD through exercise concurrently with gait and motor performance. Another limitation of the meta-analyses on the outcomes of gait capacity measures and the UPDRS-III scores is potential publication bias, which was addressed by the trim and fill method. Finally, the literature search was restricted to studies published in English and conducted only in three major electronic databases, which raises the possibility that some relevant studies may have been missed. However, we tried to reduce this possibility by manually searching the reference lists of retrieved studies to identify any additional relevant clinical trials.

Postural control and gait rely on complex anatomical and neurophysiological substrates and treatment of balance and gait disturbances in PD can prove challenging [86,87]. It is thus likely that most effective future treatments will combine physiotherapy with invasive or non-invasive neuromodulation of underlying neural circuits and/or pharmacological treatments.

Although our study provided evidence that physiotherapy interventions could show sustained effects on mobility in PD, there are still many questions that need to be answered regarding the recommended physiotherapy scheme tailored to the individual patient. Regarding the clinical implementation of the present findings, many of the interventions reviewed were intensive and delivered under close supervision. It is thus possible that effects as large as these reported here might not be realistically achieved in routine practice. Future studies should aim to investigate the long-term effects of physiotherapeutic interventions beyond the commonly assessed 3- and 6-month follow-up periods in order to clarify the duration and stability of clinical benefits. Additionally, large-scale trials with large sample size and multicenter designs are necessary to enhance the statistical power and generalizability of findings. Fortunately, such studies are already underway, and they are expected to provide valuable insights into the appropriate dosing of exercise and durability of exercise effects in PD, particularly regarding aerobic exercise [88]. The refinement of objective tools—such as wearable movement sensors and digital gait analysis—which are becoming widely available, could provide more sensitive and unbiased assessment of spatiotemporal gait parameters in future studies. Finally, the integration of biomarkers and neuroimaging techniques may provide valuable insights into the underlying pathophysiological mechanisms of Parkinson’s disease and the observed clinical improvements.

## 5. Conclusions

This systematic review and meta-analysis demonstrated that physiotherapy interventions are capable of producing sustained improvements in balance, gait, and general motor function in individuals with Parkinson’s disease, which are evident at least 3 months after treatment cessation. Multimodal exercise programs conferred the most consistent and clinically meaningful benefits, particularly when delivered over longer durations. Benefits for balance and general motor performance were observed across disease stages, whereas gait improvements were evident in early- to mid-stage disease. Our results provide a basis for informing clinical practice guidelines.

Overall, the findings support physiotherapy as a core component of Parkinson’s disease management, alongside other disciplines such as occupational therapy, with future research needed to define optimal dosing, long-term sustainability, and personalized intervention strategies.

## Figures and Tables

**Table 1:** The characteristics of studies included in the meta-analyses.

Source	Participant Characteristics	Sample Size (N)	Mean Age (±SD) (yrs)	Sex (% Male)	Intervention Group(s)	Comparison Group	Outcome Measures	Assessments and Follow-Up	Results
Aerobic Training									
Qutubuddin et al., 2013 [20]	PD Duration > 3 years Motor severity: UPDRS-III > 30 Good response to pharmacological treatment (on-medication) Average disease duration: approximately 7.2 years	Exercise group (Theracycle): 13 Control group (usual care): 10	Total: 68.2 ± 8.8	Not reported	Continuous aerobic cycling on a Theracycle, 16 sessions over 8 weeks, with 2 sessions per week.	Usual clinical care.	BBS UPDRS-III	Baseline: at the start POST: immediately after 8 weeks of exercise Follow-up: 4 months after the end of the exercise	UPDRS-III of the experimental group showed improvement 4 months post-intervention.
Picelli et al., 2013 [21]	Idiopathic PD (UKBBC) Hoehn & Yahr stage III MMSE > 24 No prior training at least 3 months before the study	Total participants: 60 Robotic gait training (RGT) group: 20 Treadmill training (TT) group: 20 Conventional physiotherapy (PT) group: 20	Total: 68.3 ± 8.3 έτη RGT: 68.50 (10.10) TT: 68.80 (7.72) PT: 67.55 (7.08)	Total: 38.3% M, 61.7% F RGT group: 9/11 (M/F) TT group: 6/14 (M/F) PT group: 8/12 (M/F)	4-week/12 sessions (45 min each, 3 times/week) The robotic-assisted group (GT1 Gait Trainer) with progressive reduction in body weight support and gradual speed increases, while the conventional treadmill group (Jog Now 500MD) trained at alternating speeds.	Conventional physiotherapy program and PNF facilitation techniques.	BBS 10MWT 6MWT Spatiotemporal gait parameters (GAITRite) UPDRS	T0: before the intervention T1: immediately after the 4-week intervention T2: 3 months after the end of the intervention	Long-term improvements were observed in RGT and TT groups at 3 months (6MWT, 10MWT and stride length). Balance (BBS) improved only in the RGT group and was maintained, while UPDRS also showed greater improvement in RGT.
Resistance Training									
Chen et al., 2021 [22]	Idiopathic PD (UKBBC) Hoehn & Yahr II–III Age 50–75 years On stable medication Ability to walk independently MMSE ≥ 24	Total: 74 Gym group (weight machines): 23 Free group (free weights, elastic bands): 25 CG (home stretching): 25 Final analyzed sample: 68	GG: 63.4 (6.9) FG: 63.2 (6.4) CG: 63.6 (7)	GC: 17 (73.9) (M) FG: 18 (69.2) (M) CG: 18 (72) (M)	GC: Biodelta machines FG: Equivalent exercises with dumbbells, elastic bands, and ankle weights. The program lasted 3 months (2 sessions/week/50 min)	Home stretching (main muscle groups, without progressive loading or resistance training).	Mini-BEST BBS Force platform TUG UPDRS-III	Baseline 1 week after the end of intervention (at 3 months) 6 months after the end of intervention	Balance (Mini-BEST & BBS) improved in the FG and was maintained at 6 months.
Morris et al., 2015 [23]	PD MMSE ≥ 24 H&Y < 5 Able to perform the interventions No DBS	Total: 210 Progressive resistance strength training (PRST): 70 Movement strategy training (MST): 69 Life skills (LS): 71	Total: 67.9 (9.6) PRST: 67.4 (10.4) MST: 68.4 (9.9) LS: 67.9 (8.4)	Total: 140/70 (M/F) PRST: 42/28 (M/F) MST: 46/23 (M/F) LS: 52/19 (M/F)	PRST: functional strength exercises + weekly program of home exercise. MST: fall prevention and strategies, independently at home. x1/week/120 min for 8 consecutive weeks.	Social and educational session.	6MWT (speed) Speed (stop watch) UPDRS-IIITUG Falls	Baseline After 8 weeks of therapy At 3 months and 12 months after the end of therapy	At 12 months, PRST and MST reduced fall rates compared to LS. No differences were observed in walking speed or TUG performance between groups.
Li et al., 2012 [24]	PD Age 40–85 years Hoehn & Yahr I–IV On stable medication MMSE ≥ 24 UPDRS-III: at least one limb with a score ≥ 2	Total: 195 Tai chi group: 65 Resistance training (RES) group: 65 Stretching (control) group: 65	Tai chi: 68 ± 9 RES: 69 ± 8 Stretching: 69 ± 9	Tai chi: 20 (30.8) (F) RES: 27 (41.5) (F) Stretching: 26 (40.0) (F)	24-week program (2 sessions/week, 60 min). Tai chi group: postural stability, weight shifting, stepping, and ankle exercises. Resistance training: muscle strengthening for posture, balance, and gait.	Gentle program of seated and standing stretches, breathing, and relaxation techniques.	Balance parameters (balance master system) FRT TUG Gait parameters (GAITRite) Fall diary	Baseline At 3 months (mid-intervention) At 6 months (end of intervention) 3 months after the completion of the intervention (follow-up)	Tai chi group had improvements in all assessed parameters and the largest fall reduction, with benefits maintained at 3-month follow-up.
Gait/Balance Training									
Capato et al., 2020 [25]	PD (UKBBC) Hoehn & Yahr I–III History of falls (past 1 year) Independent walking for 10 min MMSE ≥ 24 Independent indoor walking On stable medication No visual or hearing impairments Stable deep brain stimulation settings	Total: 154 RAS-multimodal BT: 56 Multimodal BT: 50 Control group: 48	RAS-MBT: 74 (8) MBT: 67 (13) CG: 73 (10)	RAS-MBT: 27 (48% M) MBT: 32 (64% M) CG: 29 (60% M)	40 balance and gait exercises using visual cues as part of the standard physiotherapy. The balance training group with rhythmic auditory cues (RAS) using a metronome. 5 weeks, 10 sessions/45 min each, twice a week.	General educational and fall prevention program consisting of 10 sessions, each lasting 45 min.	BBS Retropulsion test Push and release test Mini-BESTest, Rapid turns Test TUG TUG dual-task UPDRS-III FES-I NFOG-Q	Baseline Post: immediately after the intervention FU-1: 1 month after the end of the intervention FU-6: 6 months after the end of the intervention	Balance and gait gains were maintained at 1 month for both and at 6 months only for the RAS, except for the rapid turns test.
Wállen et al., 2018 [26]	Idiopathic PD Age ≥ 60 years Hoehn & Yahr 2–3 Walk indoors independently On stable medication No other severe medical conditions	Training group: 51 Control group: 49 76 remained	Training group: 73.1 (5.8) Control group: 73.0 (5.5)	Training group: 32/19 (M/F) Control group: 25/24 (M/F)	10-week HiBalance program (group balance and dual-task exercises, 3 sessions/week), targeting on sensory integration, postural control, and motor ability, followed by individualized physical activity plans.	Usual daily routine.	Mini-BESTest Gait velocity Step length, Dual-task habitual physical activity (daily steps)	Baseline Post-intervention At 6 months and 12 months	At 10 weeks, the training group improved in all measures, except daily steps and dual-task performance, but by 6–12 months, balance, gait speed, and step length returned to baseline.
Wong Yu et al., 2015 (b) [27]	See above [Wong-Yu et al., 2015 (a)] [28]	Total: 68 BAL group: 32 CON group: 36	BAL group Mean age: 60.2 ± 9.0 CON group Mean age: 61.9 ± 8.5	BAL group (M/F): 19/13 CON group (M/F): 20/16	See above [Wong-Yu et al., 2015 (a)] [28]	See above [Wong-Yu et al., 2015 (a)] [28]	Mini-BESTest FR FTSTS OLS TUG Dual-task TUG time	At 1 week pre-training (pre) Immediately post-training (post) At 6 months post-training	At 6 months post-training, the BAL group showed significantly greater improvements compared to the control group in balance (Mini-BESTest, FR, OLS) and mobility (FTST, TUG and dual-task TUG).
Tollar et al. 2019 [29]	PD Hoehn & Yahr 2–3 Mobility difficulty and postural instability Exclusion: MRI-based brain abnormalities, MMSE < 24, BDI > 40, severe cardiac disease, uncontrolled diabetes, history of stroke, traumatic brain injury, seizure disorder, DBS, vestibular/visual dysfunction, limiting locomotion or balance or current exercise program.	Total *n* = 55 E + M (exercise + maintenance) *n* = 19 E (exercise) *n* = 16 C (control) *n* = 20	Total: 67.6 ± 3.75 E + M: 67.5 ± 3.91 E: 67.6 ± 3.26 C: 67.6 ± 4.08	Total: 29 (M) E + M: 11 (M) E: 6 (M) C: 12 (M)	High-intensity and high-frequent exergaming agility program for 3 weeks (5 sessions/week). Sensorimotor/visuomotor agility training, X-box Kinect-based exergaming, focusing on balance, gait, coordination, and posture. This was followed by a 72-month maintenance program for the E + M group (3 sessions/week).	Habitual activities.	MDS-UPDRS M-EDL TUG Postural stability (3D path of the center of pressure)	Baseline (time 0) After the 3-week program At 3, 6, 9, 12, 18, and 24 months	MDS-UPDRS M-EDL improved in both intervention groups. E + M sustained these gains over two years, whereas in E were transient. TUG and postural stability improved in both E and E + M, but long-term maintenance of benefits was observed only in E + M.
Chivers-Seymour et al., 2019 [30]	PD (UKBBC) Independently mobile with or without an aid At least one fall in the previous 12 months MMSE ≥ 24 Cognitive ability to give informed consent Able to participate in program. Hoehn and Yahr stage I-IV	PDSAFE *n* = 238 Control *n* = 236	PDSAFE: 71 (7.7) Control: 73 (7.7)	PDSAFE: 147 (62%) (M), 91 (38%) (F) Control: 119 (50%) (M), 117 (50%) (F)	Usual care + PDSAFE program (balance and strength training, functional exercises, and strategies for preventing falls and freezing) for over 6 months (12 supervised/1–1.5 h and unsupervised 30 min daily).	Usual care and Parkinson’s UK DVD with information.	Falls Mini-BESTest The chair stand test (CST) FES NFoG	Baseline 0–6 months 6–12 months (6 months follow-up)	At 12 months, benefits were not maintained (near-fall rate, balance, falls confidence, functional strength in PDSAFE).
Dual-Task Training									
Da Silva, Iucksch & Israel, 2023 [31]	Idiopathic PD H&Y I–IV Medical clearance for exercise in a heated pool Ability to walk independently MMSE > 21	Total: 28 EG: 14 CG: 14 (11 in the final analysis)	EG: 63 ± 13 CG: 64 ± 13	EG: 5/8 (M/F) CG: 6/5 (M/F)	EG: dual-task training in a heated pool (progressive motor and cognitive exercises). The program lasted for over 20 sessions x2/week/60 min	CG: daily activities.	UPDRS-III Falls	AS1 (before intervention) AS2 (immediately after intervention) AS3 (3 months after AS2)	EG: improvements in UPDRS-II (ADLs) and UPDRS-III scores and were maintained at AS3.
Silva & Israel, 2019 [32]	Idiopathic PD H&Y I–IV Medical clearance for exercise in a heated pool Independent walking MMSE > 21	Total: 28 EG: 14 CG: 14 (11 in the final analysis)	EG: 63.12 ± 13.61 CG: 64.23 ± 13.45	EG: 6/5 (M/F) CG: 5/8 (M/F)	See above (Da Silva, Iucksch & Israel, 2023)	See above (Da Silva, Iucksch & Israel, 2023).	BBS DGI TUG FTSST	AS1 (before the intervention) AS2 (immediately after) AS3 (3 months after AS2)	EG: improvements in all tests compared to the CG, which were maintained at AS3.
Virtual Reality									
Mirelman et al., 2016 [33]	Independent walking for 5 min On stable medication Two or more falls in the past 6 months Mild cognitive impairment (MMSE ≥ 21) H&Y II–III	Total: 302 VR-TT (Intervention): 154 (PD = 66) TT (Control): 148 (PD = 64)	VR-TT: 74.2 (6.9) TT: 73.3 (6.4)	VR- TT: 74.2 (6.9) (M) TT: 73.3 (6.4) (M)	VR-TT: obstacle courses, multiple pathways, distractions, and real-life simulations with visual and auditory feedback. The program lasted over 6 weeks (x3/week/45 min).	TT: Similar program without virtual reality.	SPPB Gait parameters (IMUs) 2MWT Fall diaries	T0: Baseline T1: 1 week after the end of intervention T2: 1 month after the end of intervention T3: 6 months after the intervention	VR-TT: reduction in falls at 6 months post-training and a lower fall rate compared to the TT at the same point. Improvements were also observed in gait variability, step height, and walking speed.
Technology/Robot Assisted Training									
Carda et al., 2012 [34]	Idiopathic PD (UKBBC) Age < 75 years Hoehn & Yahr < III No motor fluctuations Independent walking ability No prior specific gait training in the last 6 months	Total: 30 Lokomat: 15 TT: 15 Final participants: 28 (due to dropouts)	Total: 67.89 ± 5.4 years Intervention group: 67.87 ± 7.05 years Control group: 66.93 ± 5.13 years	Total: 60.7% M (*n* = 17)	Robotic-assisted body weight-supported treadmill training (Lokomat) for over 4 weeks (3 sessions/week, 30 min), with treadmill speed progressively increased based on endurance.	Conventional treadmill gait training.	TUG 10MWT 6MWT UPDRS-III	Before the intervention, Immediately after the intervention (4 weeks) 3 months post-intervention 6 months post-intervention.	Both groups improved in the 6MWT, 10MWT, TUG, and UPDRS-III and were maintained at 6 months. The Lokomat group showed the greatest improvement at 3 months, with a slight decline at 6 months.
Furnari et al., 2017 [35]	Idiopathic PD (UKBBC) Hoehn & Yahr II–III On stable medication Independent walking ability MMSE ≥ 23 No motor fluctuations No significant cardiovascular, respiratory or other conditions affecting gait	Total: 38 EG = 19 (intervention group) CG = 19 (control group)	Total: 74.6 ± 10.2 EG: 71.5 ± 11.7 CG: 77.7 ± 8.3	Total: 17 (44.7% F) EG: 8 (42.0%) CG: 9 (47.3%)	Robotic-assisted gait training (Lokomat) with progressive increases in speed and reduced body weight support, combined with conventional physiotherapy program. The intervention lasted 4 weeks/12 sessions, 3 times per week.	Conventional physiotherapy and PNF facilitation techniques for over 4 weeks (6 times/week, 1 h per session).	Tinetti balance Tinetti walking, 10MWT UPDRS-III	T0: before the start of treatment T1: immediately after 4 weeks of treatment T2: 3 months after the end of the treatment	Long-term improvements in balance and gait were largely maintained, with a slight decline in both groups. Only the EG retained improvements in the Tinetti Walking and UPDRS-III.
Dance									
Pohl et al., 2020 [36]	Age < 18 years PD Hoehn & Yahr ≤ 3 On stable medication Ability to walk 10 m without assistance MoCA ≤ 25	Total: 46 Intervention group: 26 Control group: 20	Intervention group: 69.7 ± 7.0 years Control group: 70.4 ± 6.0 years	Intervention group: 19 M (73%) Control group: 13 M (65%)	The 12-week Ronnie Gardiner program included 60 min sessions (2/week) with 50 min of choreomotor exercises.	Usual care.	TUG Mini-BESTest FOGQ FES-I	Baseline 2 weeks after the end of the intervention 3 months after the end of the intervention	The intervention improved FES-I at 3 months, but not Mini-BESTest or TUG dual-task. Short-term gains were not maintained.
Tai Chi/Qigong									
Schmitz- Hübsch et al., 2006 [37]	PD regardless of stage and motor complications On stable medication MMSE > 24 Excluded: prior Qigong experience	Total: 56 Qigong group: 32 Control group (CG): 24	Qigong group: 64 ± 8 Control group (CG): 63 ± 8	Qigong: 24/8 (M/F) CG: 19/5 (M/F)	Qigong program (“Frolic of the Crane” and “Eight Brocades”) and home practice. 16 lessons over two 8-week cycles, with one 60 min session/week.	No other intervention.	UPDRS-III	Baseline 3 months after start 6 months after start 12 months after start of the intervention	Qigong group showed significant improvement in UPDRS-III (question 30) at 3 and 6 months, with partial maintenance at 12 months.
Pilates									
Coban et al., 2025 [38]	PD Hoehn & Yahr stage II–III Age > 45 years MMSE ≥ 24 Turkish language proficiency Diagnosis ≥ 2 years No other neurological conditions or medication changes during the study	Total: 32 Parkinson Pilates (PP) group: 18 Conventional physiotherapy (CP) group: 18	PP: 70.19 ± 8.88 CP: 72.0 ± 7.3	PP: 5 (31.25% M) CP: 9 (56.25% M)	A 12-week Parkinson Pilates program using multisensory cues, with 24 supervised sessions (2 per week, 60 min each) and 4 home practice sessions per week.	Active conventional physiotherapy program (strengthening, flexibility, and endurance exercises).	GABS NFRT BBS TUG FRT UPDRS-III	Baseline 6th week (mid-intervention) 12th week (end of intervention) 24th week (3-month follow-up)	The PP group showed lasting improvements in BBS, FRT, GABS, NFRT, and gait rhythm at 3 months, while TUG and UPDRS-III were not maintained.
Multimodal Training/Physical Therapy									
Ashburn et al., 2007 [39]	Idiopathic PD Hoehn & Yahr 2–4 Independently mobile Living at home in the community >1 fall in the previous 12 months	Total: 142 EG: 70 CG: 72	EG: 72.7 (9.6) CG: 71.6 (8.8)	EG: 38 (54% M) CG: 48 (67% M)	Strength, ROM exercises, balance training, strategies for fall prevention and movement initiation, and compensation technique, daily for 1 h at home for 6 wk. After the end, monthly follow-up phone calls were conducted to encourage their training.	Usual care.	TUG BBT FRT Chair stand test SAS Fall diaries	Baseline 8 weeks 6mo (18 weeks after the end of the program)	The EG showed lower fall and injury rates at 8 weeks and 6 months and improved FR at 6 months. No significant differences were observed in BBT, SAS, TUG, ROM, or muscle strength.
Dipasquale et al., 2017 [40]	Idiopathic PD Diagnosis ≥ 24 months Hoehn & Yahr II Medical therapy unchanged Ability to follow the study protocol	Physiotherapy group (Phys): 20 General exercise (GE): 20 Totally, 9 drop outs (4 phys and 5 GE)	Phys: 69.9 (6.42) GE: 66.4 (9.32)	Phys: 13 (65% M) GE: 13 (65% M)	Physiotherapy group followed the Royal Dutch Society for Physical Therapy guidelines. Two weekly sessions of 1 h each for 4 months.	General exercise program for PD.	FIM TUG UPDRS-III	T0: before the start of the treatment T1: st the end T2: follow-up 135 ± 20 days after the end of the treatment	The phys group showed sustained FIM improvements, decreased UPDRS-III, and better TUG and walk performance. The GE group had short-term FIM gains, increasing UPDRS-III.
Zanchet et al., 2025 [41]	PD Diagnosis Age 18- 80 On stable medication or DBS with stable stimulation parameters No severe PD or conditions limiting exercise (excluded patients presenting a Hoehn and Yahr scale > 3)	Total: 44 APA+ (adapted physical activity): 22 APA-: 22	Total: 67.7 ± 7.3 APA-: 68.1 ± 7.3 APA+: 67.3 ± 7.5	Total: 30 (68.2) (M%) APA-: 15 (68.2) (M%) APA+: 15 (68.2) (M%)	3-month APA program, with 2 sessions/week for 1 h. Aerobic, strength, and stretching exercises plus physical activities, education, and a 2-weekly physiotherapy focusing on gait and balance.	The APA- group could engage in other physical activities, as mentioned for the APA+ group.	UPDRS-III 6MWT	Baseline M3: immediately after the end of the 3-month intervention M9: 6 months after the end of the program	The UPDRS-III scores decreased in the APA+ group and increased in the APA- group by M3, with no difference at M9.
Mak et al., 2024 [13]	Age 30–80 years Idiopathic PD Modified Hoehn & Yahr 1.5–3 Ability to walk ≥ 30 m independently No “on–off” motor fluctuations MoCA ≤ 25 (indicating reduced cognitive function)	Total: 99 Balance and brisk walking (B&B) group: 49 Control group (CG): 50	B&B: 64/11 CG: 62/10	B&B: 26/23 (F/M) CG: 33/17 (F/M)	12-month, group-based program (balance, brisk walking, music, and dual-task activities). First 6 weeks (6 times/week), which gradually decreased over time.	Flexibility, strengthening and stretching exercises for 180 min per week.	TUG Dual-Task Mini-BESTest ABC Scale comfortable gait speed (CGS) 6MWT MDS-UPDRS-III Fall diary	PRE: before and at the start of the intervention POST: at 6 months (end of intervention) FU: at 12 months (6 months after the end of the intervention)	The B&B group showed lasting improvements in the MDS-UPDRS-III, Mini-BESTest, 6MWT and dual-task performance, while the ABC scale remained stable in the B&B group.
Frazzitta et al., 2015 [42]	Idiopathic PD Hoehn & Yahr 1–1.5 Walk without physical assistance MMSE ≥ 26 No serious comorbidity No vestibular/visual dysfunction limiting locomotion or balance	Total: 40 MIRT (multidisciplinary intensive rehabilitation treatment) group: 20 CG: 20 Totally, 9 drop out (4 MIRT and 5 CG)	MIRT Group: 69 ± 6 Control group: 68 ± 8	MIRT Group: 45 (% M) Control group: 60 (% M)	4-week multidisciplinary program with 3 1 h daily sessions, 5 days/week (balance, gait training with cues, and occupational therapy), with encouragement to continue exercises at home.	Pharmacological treatment.	TUG 6MWT UPDRS-III	T0: baseline T1: 6 months T2: 1 year T3: 18 months T4: 24 months	The MIRT group showed improvements immediately after, with sustained gains in UPDRS-III, TUG at T1, T3, and T4.
Avenali et al., 2021 [43]	PD (UKBBC) Hoehn & Yahr ≤ 3 PD-MCI single- or multiple- domain (level II) No other psychiatric, neurological, or severe pathological conditions On stable medication	Total: 34 (firstly 40) PT (physical therapy): 15 CT (control group): 19	Total: 72.3 ± 6.5 PT: 73.2 ± 7.1 CT: 71.6 ± 6.0	Total: 21/13 (M/F) PT: 7/8 (M/F) CT: 14/5 (M/F)	Physiotherapy program: aerobic, coordination, cognitive engagement exercises, and treadmill training. 6 sessions/60 min for 4 weeks.	Drug treatment and advice to remain physically active.	Tinetti balance and gait score MDS-UPDRS-III Hauser Index	T0: baseline T1: 4 weeks after baseline T2: 6 months after baseline (5 months after the end of the treatment)	PT improved motor performance at T2. UPDRS-III and Tinetti scores declined from T1 to T2, but improved from T0 to T2, with the intervention group showing greater UPDRS-III.
Monticone et al., 2015 [44]	Idiopathic PD Hoehn & Yahr 2.5–4 A decline in function Age > 50 years Disease duration >10 years On stable medication No other severe medical conditions	Experimental group (EG): 35 Control group (CG): 35	EG: 74.1 (6.0) CG: 73.4 (7.0)	EG: 24/11 (M/F) CG: 22/13 (M/F)	Motor training (transfers, functional activities, treadmill training, balance, and gait exercises, etc.); cognitive and ergonomic training for over 8 weeks, with daily 90- minute sessions.	Neuromotor training (joint mobilization, strength exercises, stretching, balance, and gait training) for 8 weeks.	BBS MDS-UPDRS-III FIM	Before treatment Post-treatment: 8 weeks later 1-y follow-up: 12 months after discharge	Both groups improved motor function, but EG showed greater and sustained gains, with larger improvements in BBS and FIM over time.
LSVT BIG									
Dashtipour et al., 2015 [45]	PD diagnosis (UKBBC) 30–90 years On stable medication No other medical conditions	General exercise (GE): 5 LSVT BIG: 6	Total: 63.4 GE: 64.0 (4.2) LSVT BIG: 62.8 (13.9)	F/M ratio: 6/5	LSVT BIG: large amplitude functional movements performed for 60 min over a period of 4 weeks.	General exercise protocol: treadmill training and seated upper limb exercise.	UPDRS	Baseline Immediately after the end of the program After 3 and 6 months	The UPDRS scores showed significant improvement at 6 months. Both interventions were equally effective at the 6-month follow-up.

Abbreviations: UK PDS BBC = UK Parkinson’s Disease Society Brain Bank criteria; PD = Parkinson’s Disease; MMSE = Mini-Mental State Examination; FOG = freezing of gait; FOGQ = freezing of gait questionnaire; BBS = Berg balance scale; BBT = Berg balance test; TUG = timed up and go; 10MWT = 10-m walk test; FES = falls efficacy scale; FES-I = Falls Efficacy Scale International; UPDRS-III = Unified Parkinson’s Disease Rating Scale; ABC Scale: activities-specific balance confidence scale; 6MWT = 6-min walk test; 4MWT = 4-m walk test; 2MWT = 2-min walk test; SPPB = Short Physical Performance Battery; STS = sit-to-stand test; FTSTS = five times sit-to-stand; MDS-UPDRS-III = Movement Disorder’s Society UPDRS-III; FDM-T = force distribution measurement treadmill; OLS = one-leg stand test; DGI = dynamic gait index; FWD = forward; BKD = backward; PCI = phase coordination index; EG = experimental group; CG = control group; F = females; M = males; SLS = single-leg stance; NFOGQ = New freezing of gait Questionnaire; PNF = proprioceptive neuromuscular facilitation; PTFMB = provocative test for freezing and motor blocks; GABS = Clinical Gait And Balance Scale; FRT or FR = functional reach test; NFRT = Nelson foot reaction test; SAS = self-assessment Parkinson’s disease disability scale; FIM = functional independence measure; Mini-BESTest = mini-balance evaluation systems test; RAS = rhythmic auditory stimulation; DBS = deep brain stimulation; FU = follow-up; LSVT = Lee Silverman voice treatment.

## Data Availability

The data presented in this study are available upon request from the corresponding author.

## Supplementary Materials

The following supporting information can be downloaded at: https://www.mdpi.com/article/10.3390/neurosci7020042/s1, File S1: Search strategy, Table S1: Characteristics of studies included in the review (both narrative and meta-analysis), Figure S1: Funnel plot A, Figure S2: Funnel Plot B, Figure S3: Funnel plot C.
