# Supplementary material for: Sustained Effects of Physiotherapy Interventions on Balance, Gait, and General Motor Function in Patients with Parkinson’s Disease: A Systematic Review and Meta-Analysis
Source: NeuroSci. 2026 Apr 3;7(2):42. doi: 10.3390/neurosci7020042 (PMC13119462; doi:10.3390/neurosci7020042)
Supplement: Supplementary file 1 [file neurosci-07-00042-s001.zip › File S1.pdf]

Search strategy:

PUBMED

("Parkinson's disease"[MeSH Terms] OR "Parkinson's disease" OR "parkinsonian") AND ("cognitive-motor training" OR "simultaneous task training" OR "divided attention training" OR "dual task training") AND ("balance"[MeSH Terms] OR "postural control" OR "stability") AND ("gait"[MeSH Terms] OR "walking" OR "motor function")

("Parkinson's disease"[MeSH Terms] OR "Parkinson's disease") AND ("resistance training"[MeSH Terms] OR "resistance training" OR "strength training" OR "weight training") AND ("balance"[MeSH Terms] OR "stability" OR "postural control") AND ("gait"[MeSH Terms] OR "walking" OR “motor function”)

("Parkinson's disease"[MeSH Terms] OR "Parkinson's disease") AND ("virtual reality"[MeSH Terms] OR "virtual reality" OR "exergames" OR "video games") AND ("balance"[MeSH Terms] OR "postural balance" OR "stability" OR "postural control") AND ("gait"[MeSH Terms] OR "walking" OR "motor function")

("Parkinson's disease"[MeSH Terms] OR "Parkinson's disease") AND ("aerobic exercise"[MeSH Terms] OR "aerobic exercise" OR "aerobic training" OR "endurance training") AND ("balance"[MeSH Terms] OR "postural balance" OR "stability" OR "postural control" OR "gait"[MeSH Terms] OR "walking" OR "motor function")

("Parkinson's disease"[MeSH Terms] OR "Parkinson's disease") AND ("dance therapy"[MeSH Terms] OR "dance therapy" OR "tango" OR "ballet" OR "ballroom" OR "music therapy"[MeSH Terms] OR "music therapy" OR "rhythmic auditory stimulation" OR "yoga"[MeSH Terms] OR "yoga" OR "stretching" OR "breathing practice" OR "tai chi"[MeSH Terms] OR "tai chi" OR "alternative therapy" OR "qigong" OR "pilates"[MeSH Terms] OR “pilates”) AND ("balance"[MeSH Terms] OR "postural balance" OR "stability" OR "postural control") AND ("gait"[MeSH Terms] OR "walking" OR "motor function")

("Parkinson's disease"[MeSH Terms] OR "Parkinson's disease") AND ("cueing"[MeSH Terms] OR "cues" OR "cueing" OR "cueing strategies" OR "acoustic cues" OR "sound cues" OR "auditory cues" OR "visual cues" OR "sensory cues") AND ("balance"[MeSH Terms] OR "postural balance" OR "stability" OR "postural control") AND ("gait"[MeSH Terms] OR "walking" OR "motor function")

("Parkinson's disease"[MeSH Terms] OR "Parkinson's disease") AND ("robotics"[MeSH Terms] OR "robotics" OR "robot assisted interventions" OR "technology" OR "technology interventions" OR "wearable" OR "wearable devices") AND ("balance"[MeSH Terms] OR "postural balance" OR "stability" OR "postural control") AND ("gait"[MeSH Terms] OR "walking" OR "motor function")

("Parkinson's disease"[MeSH Terms] OR "Parkinson's disease") AND ("physiotherapy"[MeSH Terms] OR "physiotherapy" OR "physical therapy" OR "exercise") AND ("balance"[MeSH

Terms] OR "postural balance" OR "stability" OR "postural control") AND ("gait"[MeSH Terms] OR "walking" OR "motor function")

("Parkinson's disease"[MeSH Terms] OR "Parkinson's disease") AND ("balance training"[MeSH Terms] OR "postural stability exercise" OR "balance exercise") AND ("balance"[MeSH Terms] OR "postural balance" OR "stability" OR "postural control") AND ("gait"[MeSH Terms] OR "walking" OR "motor function")

("Parkinson's disease"[MeSH Terms] OR "Parkinson's disease") AND ("gait training"[MeSH Terms] OR "walking training" OR "gait exercise") AND ("balance"[MeSH Terms] OR "postural balance" OR "stability" OR "postural control") AND ("gait"[MeSH Terms] OR "walking" OR "motor function")

Additional search refinement: Following the initial search, the strategy was refined by adding the terms ("long term effects"[MeSH Terms] OR "long term effects" OR "sustained effects") using the Boolean operator AND, in order to identify studies reporting long-term outcomes

SCOPUS

("Parkinson's disease"[MeSH Terms] OR "Parkinson's disease") AND ("cognitive-motor training"[MeSH Terms] OR "cognitive-motor training" OR "simultaneous task training" OR "divided attention training" OR "dual task training")
AND ("balance"[MeSH Terms] OR "postural balance" OR "stability" OR "postural control") AND ("gait"[MeSH Terms] OR "walking" OR "motor function")

("Parkinson's disease"[MeSH Terms] OR "Parkinson's disease") AND ("resistance training"[MeSH Terms] OR "resistance training" OR "strength training" OR "strength exercise" OR "weight training") AND ("balance"[MeSH Terms] OR "postural balance" OR "stability" OR "postural control") AND ("gait"[MeSH Terms] OR "walking" OR "motor function")

("Parkinson's disease"[MeSH Terms] OR "Parkinson's disease") AND ("virtual reality"[MeSH Terms] OR "virtual reality" OR "exergames" OR "video games" OR "gaming") AND ("balance"[MeSH Terms] OR "postural balance" OR "stability" OR "postural control") AND ("gait"[MeSH Terms] OR "walking" OR "motor function")

("Parkinson's disease"[MeSH Terms] OR "Parkinson's disease") AND ("aerobic exercise"[MeSH Terms] OR "aerobic exercise" OR "aerobic training" OR "endurance training") AND ("balance"[MeSH Terms] OR "postural balance" OR "stability" OR "postural control" OR "gait"[MeSH Terms] OR "walking" OR "motor function")

("Parkinson's disease"[MeSH Terms] OR "Parkinson's disease") AND ("dance therapy"[MeSH Terms] OR "dance therapy" OR "tango" OR "ballet" OR "ballroom" OR "music therapy"[MeSH Terms] OR "music therapy" OR "rhythmic auditory stimulation" OR "yoga"[MeSH Terms] OR "yoga" OR "stretching and breathing practice" OR "tai chi"[MeSH Terms] OR "tai chi" OR "alternative therapy" OR "qigong" OR "pilates"[MeSH Terms] OR “pilates”) AND ("balance"[MeSH Terms] OR "postural balance" OR "postural stability" OR "stability" OR "postural control") AND ("gait"[MeSH Terms] OR "walking" OR "motor function")

("Parkinson's disease"[MeSH Terms] OR "Parkinson's disease") AND ("cueing"[MeSH Terms] OR "cueing" OR "cues" OR "cueing strategies" OR "acoustic cues" OR "sound cues" OR "auditory cues" OR "visual cues" OR "sensory cues") AND ("balance"[MeSH Terms] OR "postural balance" OR "stability" OR "postural control") AND ("gait"[MeSH Terms] OR "walking" OR "motor function")

("Parkinson's disease"[MeSH Terms] OR "Parkinson's disease") AND ("robotics"[MeSH Terms] OR "robotics" OR "robot assisted interventions" OR "technology"[MeSH Terms] OR "technology" OR "technology interventions" OR "wearable" OR "wearable devices") AND ("balance"[MeSH Terms] OR "postural balance" OR "stability" OR "postural control") AND ("gait"[MeSH Terms] OR "walking" OR "motor function")
("Parkinson's disease"[MeSH Terms] OR "Parkinson's disease") AND ("physiotherapy"[MeSH Terms] OR "physiotherapy" OR "physical therapy" OR "exercise") AND ("balance"[MeSH Terms] OR "postural balance" OR "stability" OR "postural control") AND ("gait"[MeSH Terms] OR "walking" OR "motor function")

("Parkinson's disease"[MeSH Terms] OR "Parkinson's disease") AND ("balance training"[MeSH Terms] OR "postural stability exercise" OR "balance exercise") AND ("balance"[MeSH Terms] OR "postural balance" OR "stability" OR "postural control") AND ("gait"[MeSH Terms] OR "walking" OR "motor function")

("Parkinson's disease"[MeSH Terms] OR "Parkinson's disease") AND ("gait training"[MeSH Terms] OR "walking training" OR "gait exercise") AND ("balance"[MeSH Terms] OR "postural balance" OR "stability" OR "postural control") AND ("gait"[MeSH Terms] OR "walking" OR "motor function")

Additional search refinement: Following the initial search, the strategy was refined by adding the terms ("long term effects"[MeSH Terms] OR "long term effects" OR "sustained effects") using the Boolean operator AND, in order to identify studies reporting long-term outcomes

<u>COCHRANE LIBRARY</u>

("Parkinson's disease"[MeSH Terms] OR "Parkinson's disease") AND ("cognitive-motor training"[MeSH Terms] OR "cognitive-motor training" OR "dual task training" OR "simultaneous task training" OR "divided attention training") AND ("balance"[MeSH Terms] OR "postural balance" OR "stability" OR "postural control") AND ("gait"[MeSH Terms] OR "walking" OR "motor function")

("Parkinson's disease"[MeSH Terms] OR "Parkinson's disease") AND ("resistance training"[MeSH Terms] OR "resistance training" OR "strength training" OR "strength exercise" OR "weight training") AND ("balance"[MeSH Terms] OR "postural balance" OR "stability" OR "postural control") AND ("gait"[MeSH Terms] OR "walking" OR "motor function")

("Parkinson's disease"[MeSH Terms] OR "Parkinson's disease") AND ("virtual reality"[MeSH Terms] OR "virtual reality" OR "exergames" OR "exergaming" OR "video games" OR

"gaming") AND ("balance"[MeSH Terms] OR "postural balance" OR "stability" OR "postural control") AND ("gait"[MeSH Terms] OR "walking" OR "motor function")

("Parkinson's disease"[MeSH Terms] OR "Parkinson's disease") AND ("aerobic exercise"[MeSH Terms] OR "aerobic exercise" OR "aerobic training" OR "endurance training") AND ("balance"[MeSH Terms] OR "postural balance" OR "stability" OR "postural control" OR "gait"[MeSH Terms] OR "walking" OR "motor function")

("Parkinson's disease"[MeSH Terms] OR "Parkinson's disease") AND ("dance therapy"[MeSH Terms] OR "dance therapy" OR "tango" OR "ballet" OR "ballroom" OR "music therapy"[MeSH Terms] OR "music therapy" OR "rhythmic auditory stimulation" OR "yoga"[MeSH Terms] OR "yoga" OR "stretching and breathing practice" OR "tai chi"[MeSH Terms] OR "tai chi" OR "alternative therapy" OR "qigong" OR "pilates"[MeSH Terms] OR pilates) AND ("balance"[MeSH Terms] OR "postural balance" OR "postural stability" OR "stability" OR "postural control") AND ("gait"[MeSH Terms] OR "walking" OR "motor function")

("Parkinson's disease"[MeSH Terms] OR "Parkinson's disease") AND ("cueing"[MeSH Terms] OR "cueing" OR "cues" OR "cueing strategies" OR "acoustic cues" OR "sound cues" OR "auditory cues" OR "visual cues" OR "sensory cues") AND ("balance"[MeSH Terms] OR "postural balance" OR "stability" OR "postural control") AND ("gait"[MeSH Terms] OR "walking" OR "motor function")

("Parkinson's disease"[MeSH Terms] OR "Parkinson's disease") AND ("robotics"[MeSH Terms] OR "robotics" OR "robot assisted interventions" OR "technology"[MeSH Terms] OR "technology" OR "technology interventions" OR "wearable" OR "wearable devices") AND ("balance"[MeSH Terms] OR "postural balance" OR "stability" OR "postural control") AND ("gait"[MeSH Terms] OR "walking" OR "motor function")

("Parkinson's disease"[MeSH Terms] OR "Parkinson's disease") AND ("physiotherapy"[MeSH Terms] OR "physiotherapy" OR "physical therapy" OR "exercise") AND ("balance"[MeSH Terms] OR "postural balance" OR "stability" OR "postural control") AND ("gait"[MeSH Terms] OR "walking" OR "motor function")

("Parkinson's disease"[MeSH Terms] OR "Parkinson's disease") AND ("balance training"[MeSH Terms] OR "postural stability exercise" OR "balance exercise") AND ("balance"[MeSH Terms] OR "postural balance" OR "stability" OR "postural control") AND ("gait"[MeSH Terms] OR "walking" OR "motor function")

("Parkinson's disease"[MeSH Terms] OR "Parkinson's disease") AND ("gait training"[MeSH Terms] OR "walking training" OR "gait exercise") AND ("balance"[MeSH Terms] OR "postural balance" OR "stability" OR "postural control") AND ("gait"[MeSH Terms] OR "walking" OR "motor function")

Additional search refinement: Following the initial search, the strategy was refined by adding the terms ("long term effects"[MeSH Terms] OR "long term effects" OR "sustained effects") using the Boolean operator AND, in order to identify studies reporting long-term outcomes
