# Supplementary material for: Sustained Effects of Physiotherapy Interventions on Balance, Gait, and General Motor Function in Patients with Parkinson’s Disease: A Systematic Review and Meta-Analysis
Source: NeuroSci. 2026 Apr 3;7(2):42. doi: 10.3390/neurosci7020042 (PMC13119462; doi:10.3390/neurosci7020042)
Supplement: Supplementary file 1 [file neurosci-07-00042-s001.zip › Table S1.pdf]

Table S1. Characteristics of studies included in the review (both narrative and meta-analysis)

Source	Participant characteristics	Sample Size (N)	Mean age (±SD) (yrs)	Sex (% male)	Intervention Group(s)	Comparison group	Outcome measures	Assessments and Follow-up	Results
Aerobic Training									
Pelosin et al., 2017 [57]	Idiopathic PD (UK PDS BBC)<br>Hoehn & Yahr stage 1–2.5<br>On stable medication<br>Ability to walk independently for 6 minutes without assistance<br>MMSE > 24<br>No FOG	Total: 30<br>HFG (High-Frequency Group): 10<br>IFG (Intermediate-Frequency Group): 10<br>LFG (Low-Frequency Group): 10	HFG: 69.9 ± 4.5<br>IFG: 73.7 ± 8.3<br>LFG: 73.1 ± 6.8	Not reported	Three groups performed treadmill gait training at low, moderate and high aerobic intensities, with the low- frequency group completing 2 sessions per week, the moderate 3 sessions per week and the high 5 sessions per week.	None	BBS<br>TUG<br>10MWT<br>FES<br>falls diary	PRE: before the start of the intervention<br>POST: immediately after the end of the intervention<br>FU-m2: 2 months after completion of the intervention<br>FU-m4: 4 months after completion of the intervention	At 4 months, the moderate- and low-frequency groups maintained or further improved their outcomes, whereas the high-frequency group showed a decline in 10MWT and FES. BBS scores improved in all groups.
Qutubuddin et al., 2013 [20]	PD<br>duration > 3 years<br>Motor severity: UPDRS-III > 30<br>Good response to pharmacological treatment (on-medication)<br>Average disease duration: approximately 7.2 years	Exercise group (Theracycle): 13<br>Control group (usual care): 10	Total: 68.2 ± 8.8	Not reported	16 sessions over 8 weeks, with 2 sessions per week<br>Warm-up, 30 minutes of continuous aerobic cycling on a Theracycle at 61-80% of HRmax (Karvonen method) followed by cool-down.	Usual clinical care without specialized physiotherapy or an exercise program.	BBS<br>UPDRS-III	Baseline: at the start<br>POST: immediately after 8 weeks of exercise<br>Follow-up: 4 months after the end of the exercise	UPDRS-III of the experimental group showed improvement 4 months post-intervention.
Rawson et al., 2019 [46]	Idiopathic PD (UKBBC)<br>Age ≥ 30 years<br>Hoehn & Yahr stage 1–4<br>Independent walking or with an assistive device for at least 3 meters<br>Good response to Levodopa<br>No dementia or vestibular disorders	Total: 96<br>Tango group (Tango): 39<br>Treadmill group (Treadmill): 31<br>Stretching group (Stretching): 26	Total: 67.16 (8.94)<br>Tango: 66.73 (9.52)<br>Treadmill 68.52 (9.54)<br>Stretching:66.18 (7.30)	Total: 40 (41.7% F)<br>Tango group: 14 (35.9% F)<br>Treadmill group: 14 (45.2% F)<br>Stretching group: 12 (46.2% F)	Tango and treadmill training: 12-week program, 2 sessions per week, 60 minutes each.	Stretching group: light stretching and flexibility exercises.	ABC Scale, Mini-BESTest<br>6MWT<br>MDS–UPDRS-III,<br>FWD gait velocity,<br>BKD gait velocity, (GAITRite)	Baseline: at the start<br>POST: immediately after the program (12 weeks)<br>Follow-up: 12 weeks after the end of the intervention	Forward velocity and backward velocity improved for the treadmill group from baseline to post-test and improvements persisted at follow-up.

Wroblewska et a., 2019 [47]	Idiopathic PD (UKBBC) Hoehn & Yahr II–III FoG in the ON state On stable medication No prior experience with Nordic Walking or supervised physiotherapy/exercise in the last 4 months	Total: 40 Nordic Walking (NW) group: 20 Control group (CG): 20	Overall mean age: 69.8 ± 7.3 NW group (Nordic Walk): 72.1 ± 7.5 CG (Control Group): 67.6 ± 6.6	Total M: NW: 12/20 CG: 9/20	12-week Nordic Walking program (24 sessions/ twice a week, 60 minutes). Three familiarization sessions preceded the intervention.	Usual care.	TUG, FOGQ, PTFMB	Baseline POST: immediately after 12 weeks Follow-up: 3 months after completion of the intervention	Nordic Walking significantly reduced FOG and improved TUG, with maintenance of gains at 3 months.
Stuckenschneider et al., 2015 [48]	Idiopathic PD Hoehn & Yahr stage 2.5–4 Absence of serious comorbidities On stable medication	Total: 22 AAE (Active Assistive Forced Exercise group): 10 Control Group (CG): initially 12 Later, the CG was converted to the PFE (Passive Forced Exercise group) with modifications: 10	Total: 71.3 ± 4.93 years AAE: 71 ± 4.62 years PFE: 71.5 ± 5.68 years CG: 71.4 ± 4.94 years	AAE: 6/4 (F/M) P: 6/4 (F/M) CG: 7/5 (F/M)	Active Assisted Forced Exercise (AAE) and Passive Forced Exercise (PFE). 12-week program using a MOTOmed stationary bicycle. AAE: supervised group PFE: individually, with passive machine- driven movement. Sessions were 3 times/week, 40 minutes each.	The PFE group initially served as the control group, receiving usual care.	Spatiotemporal gait parameters FDM-T UPDRS-III	Pre-test: before the start of the intervention Post-test: 1 day after the completion of the intervention Follow-up: 12 weeks after the end to assess maintenance of results	The AAE group improved and largely maintained gait parameters, with motor tremor gains. The PFE group showed improvement but did not maintain step length. UPDRS-III did not improve..
Picelli et al., 2013 [21]	Idiopathic PD (UKBBC) Hoehn & Yahr stage III MMSE > 24 No prior training at least 3 months before the study	Total participants: 60 Robotic Gait Training (RGT) group: 20 Treadmill Training (TT) group: 20 Conventional Physiotherapy (PT) group: 20	Total: 68.3 ± 8.3 έτη RGT: 68.50 (10.10) TT: 68.80 (7.72) PT: 67.55 (7.08)	Total: 38.3% M, 61.7% F RGT group: 9/11 (M/F) TT group: 6/14 (M/F) PT group: 8/12 (M/F)	Two groups (RGT and TT) 4-week/ 12 sessions (45 minutes each, 3 times/week) The robotic- assisted group (GT1 Gait Trainer) with progressive reduction of body weight support and gradual speed increases, while the conventional treadmill group (Jog Now 500MD) trained at alternating speeds.	Conventional physiotherapy program and PNF facilitation techniques.	BBS 10MWT 6MWT Spatiotemporal gait parameters (GAITRite) UPDRS	T0: before the intervention T1: immediately after the 4-week intervention T2: 3 months after the end of the intervention	Long-term improvements were observed in both the RGT and TT group, at 3 months (6MWT, 10MWT and stride length). Balance (BBS) improved only in the RGT group and was maintained, while UPDRS also showed greater improvement in RGT.
Resistance Training									
Chen et al., 2021 [22]	Idiopathic PD (UK Brain Bank Criteria) Hoehn & Yahr II–III Age 50–75 years	Total: 74 Gym group (weight machines): 23	GG: 63.4 (6.9) FG: 63.2 (6.4) CG: 63.6 (7)	GC: 17 (73.9) (M) FG: 18 (69.2)	GC: Biodelta machines) (e.g. chest pulls, bench presses etc.). FG: Equivalent exercises	Home stretching from standing and seated posi-	Mini-BEST BBS Force platform TUG UPDRS-III	Baseline 1 week after the end of intervention (at 3 months)	Both groups showed improvement in UPDRS-III at 3 months. Balance (Mini-BEST &

	On stable medication<br>Ability to walk independently<br>MMSE ≥ 24	Free group (free weights, elastic bands): 25<br>CG (home stretching): 25<br>Final analyzed sample: 68		(M)<br>CG: 18 (72) (M)	with dumbbells, elastic bands and ankle weights. The program lasted 3 months (2 sessions/week/50 min)	tions (main muscle groups, without progressive loading or resistance training)		6 months after the end of intervention	BBS) improved in the FG and was maintained at 6 months.
Schlenstedt et al., 2015 [58]	Idiopathic PD (UKBBC)<br>Postural instability (FAB ≤ 25)<br>Able to follow exercise<br>Exclusion: DBS, other diseases, participation in a specific RT or BT program or other medical, behavioral or exercise treatment, unstable medication	Resistance Training (RT): 17<br>Balance Training (BT): 15	RT: 75.7 ± 5.5<br>BT: 75.7 ± 7.2	RT: 5 (29.4%) (F)<br>BT: 6 (40%) (F)	RT: Exercises for hip , ankle and knee muscles, with moderate intensity and increasing resistance BT: Feedforward and feedback postural control dynamic training<br>The program lasted 7 weeks (2x/week, 60 minutes)	None	Center of mass (COM)<br>TUG<br>Gait velocity<br>Contact times (heel strike, toe off)<br>Spatio-temporal variables PCI<br>UPDRS	Baseline<br>8- weeks<br>12- weeks	Positive short-term effects were observed,but were not maintained.
Morris et al., 2015 [23]	PD<br>MMSE ≥ 24<br>H&Y <5<br>Able to perform the interventions<br>No DBS	Total: 210<br>Progressive Resistance Strength Training (PRST): 70<br>Movement Strategy Training (MST): 69<br>Life Skills (LS): 71	Total: 67.9 (9.6)<br>PRST: 67.4 (10.4)<br>MST: 68.4 (9.9)<br>LS: 67.9 (8.4)	Total: 140/70 (M/F)<br>PRST: 42/28 (M/F)<br>MST: 46/23 (M/F)<br>LS: 52/19 (M/F)	PRST: functional strength exercises + weekly program of home exercise.<br>MST: fall prevention and strategies such as attention to movement etc., independently at home. The programs consisted of x1/week/120min for 8 consecutive weeks.	Social and educational session.	6MWT (speed)<br>Speed (stop watch)<br>UPDRS-IIITUG<br>Falls	Baseline<br>After 8 weeks of therapy<br>At 3months and 12 months after the end of therapy	At 12-months, PRST and MST reduced fall rates compared to LS. No differences were observed in walking speed or TUG performance between groups.
Li et al., 2012 [24]	PD<br>Age 40–85 years<br>Hoehn & Yahr I–IV<br>On stable medication<br>MMSE ≥ 24<br>UPDRS-III: at least one limb with a score ≥ 2	Total: 195<br>Tai Chi group: 65<br>Resistance training (RES) group: 65<br>Stretching (control) group: 65	Tai Chi: 68 ± 9<br>RES: 69 ± 8<br>Stretching: 69 ± 9	Tai Chi: 20 (30.8) (F)<br>RES: 27 (41.5) (F)<br>Stretching: 26 (40.0) (F)	24-week programs (2 sessions/ week, 60 minutes).<br>Tai Chi group: postural stability, weight shifting, stepping and ankle exercises.<br>Resistance training: muscle strengthening for posture, balance and gait.	Gentle program of seated and standing stretches, breathing and relaxation techniques.	Balance parameters (Balance Master System)<br>FRT<br>TUG<br>Gait parameters (GAITRite)<br>Falls diary	Baseline<br>At 3 months (mid-intervention)<br>At 6 months (end of intervention)<br>3 months after the completion of the intervention (follow-up)	Tai Chi group led to the greatest improvements in all assessed parameters and the largest fall reduction, with benefits maintained at 3-month follow-up.
Gait/ Balance Training									

Capato et al., 2020 [25]	PD (UKBBC)<br>Hoehn & Yahr I–III<br>History of falls (past 1 year)<br>Independent walking for 10 minutes<br>MMSE ≥ 24<br>Independent indoor walking<br>On stable medication<br>No visual or hearing impairments<br>Stable deep brain stimulation settings	Total: 154<br>RAS-multimodal BT: 56<br>Multimodal BT: 50<br>Control group: 48	RAS-MBT: 74 (8)<br>MBT: 67 (13)<br>CG: 73 (10)	RAS-MBT: 27 (48% M)<br>MBT: 32 (64% M)<br>CG: 29 (60% M	40 balance and gait exercises using visual cues as part of the standard physiotherapy.<br>The balance training group with rhythmic auditory cues (RAS) used a metronome.<br>The program lasted over 5 weeks, with 10 sessions of 45 minutes each, twice a week	General educational and fall prevention program consisting of 10 sessions, each lasting 45 minutes.	BBS<br>Retropulsion Test<br>Push and Release Test<br>Mini BESTest,<br>Rapid Turns Test<br>TUG<br>TUG Dual Task<br>UPDRS-III<br>FES-I<br>NFOG-Q	Baseline<br>Post: Immediately after the intervention<br>FU-1: 1 month after the end of the intervention<br>FU-6: 6 months after the end of the intervention	Both intervention groups improved balance post-intervention, with RAS showing superior gains and FES-I improvement. Balance and gait gains were maintained at 1 month for both and at 6 months only for the RAS, except for the Rapid Turns Test.
Wállen et al., 2018 [26]	Idiopathic PD<br>Age ≥ 60 years<br>Hoehn & Yahr 2-3<br>Walk indoors independently<br>On stable medication<br>No other severe medical conditions	Training group: 51<br>Control group: 49<br>76 remained	Training group: 73.1 (5.8)<br>Control group: 73.0 (5.5)	Training group: 32/19 (M/F)<br>Control group: 25/24 (M/F)	The training group completed the 10-week HiBalance program (group balance and dual task exercises 3 sessions/week), targeting on sensory integration, postural control, and motor ability, followed by individualized physical activity plans.	Usual daily routine	Mini- BESTest<br>Gait velocity<br>Step length, Dual-task<br>Habitual physical activity (Daily steps)	Baseline<br>Post-intervention<br>At 6 months and 12 months	At 10 weeks, the training group improved in all measures except daily steps and dual-task performance, but by 6-12 months, balance, gait speed and step length returned to baseline.
Sparrow et al., 2016 [49]	Idiopathic PD<br>Hoehn & Yahr 2 -3<br>Excluded: atypical Parkinsonism, a MMSE < 26, previous surgical management of PD, or serious comorbidities that may interfere with ability to participate in the exercise program	Total: 23 – 16 completed<br>Group 1: week 0-12: active period 12 (n=12) week 12- 24: inactive period (n=7)<br>Group 2: week 0-12: inactive period (n=11) week 12- 24: active	n=16<br>Mean age 66.7 ± 5.7	n=16<br>Males: 10 (62.5%)<br>Females: 6 (37.5%)	Active period: 90-minute sessions twice weekly - active balance exercises (strengthening, ROM, anticipatory and reactive balance activities, altering sensory input and gait training).	Inactive period: did not receive intervention	Mini-BESTest<br>MDS- UPDRS III<br>FES-I	Baseline<br>12 weeks<br>24 weeks	Significant decline in the fall rate per month and improvements in balance and fear of falling.
Wong Yu et al.,	PD<br>No falls or 1 fall in the past 6 months	Total: (n=84)<br>Experimental group (EXP): 41	EXP (n=41)<br>Mean age 59.4 +-9.0	Total males: 46<br>Total females: 34	Eight-weeks group training (2h/week) included postural re-education, flexibility,	Upper limb training, postural re-	36-item BESTest<br>ABC Scale	Baseline assessment	Long-term effects:<br>6-month follow-up:<br>-Significantly greater

2015 (a) [28]	Stable medications Ability to walk independently for 30m or without assistive device Without any other neurological conditions, neurosurgery, musculoskeletal or cardiopulmonary diseases, communication or cognitive deficits MMSE < 24	Control group (CON): 43 Post training assessment analysis: 39 6-month and 12-month follow-up assessment analysis: 39	CON (n=39) Mean age 62.6 +-8.9	EXP (n=41) M/F: 25/16 CON (n=39) M/F: 21/18	strength, functional tasks, balance dance and square stepping exercise. Phase two involved 4 weeks of outdoor training with functional training and advanced balance exercises, dual-task gait training and home practice via hands-out and DVD for three hours per week during both training and follow-up period.	education, flexibility and strengthening, dexterity training. Hands-out and DVD for practicing the taught exercises for three hours per week during both training and follow-up period	Comfortable gait speed Dual-task TUG	Post-training assessment 6-month follow-up assessment 12-month follow-up assessment	increases in the BESTest total score -Significant differences over baseline values: BESTest total and subsection scores (except subsection V).
Wong Yu et al., 2015 (b) [27]	See above [Wong-Yu et al., 2015 (a)]	Total: 68 BAL group: 32 CON group: 36	BAL group Mean age: 60.2±9.0 CON group Mean age: 61.9±8.5	BAL group (M/F): 19/13 CON group (M/F): 20/16	See above [Wong-Yu et al., 2015 (a)]	See above [Wong-Yu et al., 2015 (a)]	Mini-BESTest FR FTSTS OLS TUG Dual-task TUG time	At 1 week pre-training (Pre) Immediately post training (Post) At 6 months post training	At 6 months post-training, the BAL group showed significantly greater improvements than the control group in balance (Mini-BESTest, FR, OLS) and mobility (FTST, TUG and dual-task TUG).
Terra et al., 2020 [59]	PD (UKBBC) Modified Hoehn & Yahr scale 1.5-3 Absence of cognitive deficit, characterized by the Mini Mental State Examination and other neurological or musculoskeletal disorders that could interfere in the individual's assessment or treatment	Physiotherapy + Cognitive training (PCG): 28 Motor physiotherapy (PG): 26	PCG Mean age: 67.11 ± 8.14 PG Mean age: 64.33 ± 7.77	PCG M/F: 10 (35.7%) / 18 (64.3%) PG M/F: 12 (46.2%) / 14 (53.8%)	A 4-month program (32 sessions, twice weekly) consisting of 60-minute physiotherapy sessions focused on balance, sensory integration, agility, postural control, functional independence and gait with progressively increased exercise complexity.	A 4-month program (32 sessions, twice weekly) following the same physiotherapy protocol as PG, with an additional 30 minutes per session of cognitive stimulation targeting executive, visuospatial, attentional	BESTest - balance outcome: Section I: Biomechanical constraints Section II: Stability limits/Verticality Section III: Anticipatory postural adjustments Section IV: Postural responses Section V: Sensory orientation	Pre- intervention Post-intervention Follow up (3 months)	PCG training was not superior to motor treatment in individuals with PD Evaluation after the follow-up: improvements in activities of daily living and balance maintained in PCG and only the improvement in balance was maintained.

						and motor planning functions.	Section VI: Stability in gait<br>UPDRS		
Rennie et al., 2021 [50]	Idiopathic PD (UKBBC)<br>Hoehn & Yahr 2<br>Excluded: atypical parkinsonism, a MMSE≤ 24, or had other existing neuromuscular disorders or medical conditions that influenced their gait and balance function	Total Sample (n=100)<br>Training group (n=51)<br>Control group (n=49)	Training group<br>Mean age: 73.1 ± 5.8<br>Control group<br>Mean age: 73.0 ± 5.5	Total: Males (n=56)<br>Training group: M: 32 (63%) F: 19 (37%)<br>Control group: M: 25 (51%) F: 24 (49%)	The training group completed the 10-week HiBalance program (3 sessions/week, 60 minutes, groups of 4-7) focusing on gait and balance through sensory integration, postural control, motor agility, stability limits and progressive motor-cognitive dual-tasking, followed by individualized physical activity.	Usual care	Pace (step velocity, step length, swing time variability)<br>Rhythm (step time, swing time, stance time)<br>Variability (step velocity variability, step length var., step time var., stance time var.)<br>Asymmetry (swing time asymm., step time asymm., stance time asymm.)<br>Postural control (step length asymm., mean step width, step width variability)	Baseline<br>Post intervention<br>At the 6- and 12-month follow-up	The training group showed immediate post-training improvements in gait speed, step length, swing and stance times, double support and gait variability compared to controls. However, none of these gains were maintained at 6- or 12-month follow-up.
Tollar et al 2019 [29]	PD<br>Hoehn & Yahr 2-3<br>Mobility<br>difficulty and postural instability<br>Exclusion: MRI-based brain abnormalities, MMSE<24, BDI>40, severe cardiac disease, uncontrolled diabetes, history of stroke, traumatic brain injury, seizure disorder, DBS, vestibular/visual	Total n=55<br>E+M (Exercise + Maintenance) n=19<br>E (Exercise) n=16<br>C (Control) n=20	Total: 67.6 ± 3.75<br>E+M: 67.5 ± 3.91<br>E: 67.6 ± 3.26<br>C: 67.6 ± 4.08	Total: 29 (M)<br>E+M: 11 (M)<br>E: 6 (M)<br>C: 12 (M)	High-intensity and high-frequent exergaming agility program for 3 weeks (5 sessions/week). It included sensorimotor/visuomotor agility training, X-box Kinect-based exergaming, focusing on balance, gait, coordination and posture. This was followed by a 72-month<br>maintenance program for the E+M group (3 sessions/week).	Habitual activities	MDS-UPDRS M-EDL<br>TUG<br>Postural stability (3D path of the center of pressure)	Baseline (time 0)<br>After the 3-week program<br>At 3,6,9,12,18,24 months	MDS-UPDRS M-EDL improved in both intervention groups. E+M sustained these gains over two years, whereas in E were transient. TUG and postural stability improved in both E and E+M, but long-term maintenance of benefits was observed only in E+M.

	dysfunction, limiting locomotion or balance or current exercise program.								
Hortobágyi et al 2021 [60]	See above [Tollar et al, 2019]	Total n=67<br>E+M (Exercise + Maintenance) n=19<br>E (Exercise) n=22<br>C (Control) n=26	Total: 67.8 ± 3.71<br>E+M: 67.5 ± 3.91<br>E: 68.1 ± 3.53<br>C: 67.8 ± 3.84	Total: 35 (M)<br>E+M: 11 (M)<br>E: 9 (M)<br>C: 15 (M)	See above [Tollar et al, 2019]	See above [Tollar et al, 2019]	TUG<br>MD-UPDRS M-EDL<br>Postural stability (3D path of the center of pressure)	Baseline<br>Immediate after the program<br>At 3,6,12,18,24,36,48,60,72 months	Both E and E+M groups improved M-EDL, TUG and postural stability. E+M showed additional improvement at 3 months and sustained or enhanced benefits through 72 months, whereas improvements in E lasted up to 12 months and diminished over time.
Chivers-Seymour et al., 2019 [30]	PD (UKBBC)<br>Independently mobile with or without an aid<br>At least one fall in the previous 12 months<br>MMSE ≥24<br>Cognitive ability to give informed consent<br>Able to participate in program.<br>Hoehn and Yahr stage I-IV	PDSAFE n=238<br>Control n=236	PDSAFE: 71 (7.7)<br>Control: 73 (7.7)	PDSAFE: 147 (62%)(M), 91 (38%)(F)<br>Control: 119 (50%)(M), 117 (50%)(F)	Usual care<br>PDSAFE program (balance and strength training, functional exercises and strategies to prevent falls and freezing) an individually tailored, progressive home-based exercise for over 6 months (12 supervised/ 1-1.5 hours and unsupervised 30 minutes daily).	Usual care and Parkinson's UK DVD with information.	Falls<br>Mini-BESTest<br>The chair stand test (CST)<br>FES<br>NFoG	Baseline<br>0-6 months<br>6-12 months (6 months follow-up)	At 6 months, there was a significant reduction in near-fall rate, improved balance, greater falls confidence and better balance/functional strength in the PDSAFE group. At 12 months, these benefits were not maintained.
Morris et al., 2015 [23]	See above under resistance training								
Dual Task Training									
Da Silva, Iucksch & Israel,	Idiopathic PD<br>H&Y I–IV<br>Medical clearance for exercise in a heated pool	Total: 28<br>EG: 14<br>CG: 14 (11 in the final analysis)	EG: 63 ± 13<br>CG: 64 ± 13	EG: 5/8 (M/F)<br>CG: 6/5 (M/F)	EG: dual- task training in a heated pool (progressive motor & cognitive exercises).	CG: daily activities without	UPDRS-III<br>Falls	AS1 (before intervention)<br>AS2 (immediately after intervention)	EG: improvements in UPDRS-II (ADLs) and UPDRS-III scores and were maintained at AS3.

2023<br>[31]	Ability to walk independently<br>MMSE > 21				The program lasted for over 20 sessions x2/week/60 min			AS3 (3 months after AS2)	
Silva & Israel, 2019<br>[32]	Idiopathic PD H&Y I–IV<br>Medical clearance for exercise in a heated pool<br>Independent walking<br>MMSE > 21	Total: 28<br>EG: 14<br>CG: 14 (11 in the final analysis)	EG: 63.12 ± 13.61<br>CG: 64.23 ± 13.45	EG: 6/5 (M/F)<br>CG: 5/8 (M/F)	See above (Da Silva, Iucksch & Israel, 2023)	See above (Da Silva, Iucksch & Israel, 2023)	BBS<br>DGI<br>TUG<br>FTSST	AS1 (before the intervention)<br>AS2 (immediately after)<br>AS3 (3 months after AS2)	EG: improvements in all tests compared to the CG, which were maintained at AS3.
Geroin et al., 2018 [61]	Idiopathic PD (UKBBC)<br>H& II–III<br>Independent walking for 10 minutes<br>MMSE ≥ 24<br>On stable medication<br>No hearing or vision problems<br>No unstable medical conditions affecting gait	Total: 121<br>CCT (Consecutive task training): 65<br>IDT (Integrated Dual- task Training): 56	Total: 65.93 (±9.22)<br>CCT: 66.05 (±9.30)<br>IDT: 65.80 (9.19)			CCT : sequential-task training (15 min of walking exercises, 15 min of seated cognitive exercises and 10 min of functional training with tasks practiced separately)	Spatiotemporal gait parameters (GAITRite walkway system),<br>Stroop Test<br>Backward digit span	Test 1: at baseline<br>Test 2: at the end of baseline measurements<br>Test 3: immediately after the 6-week intervention<br>Test 4: 12 weeks later (follow-up)	IDT & CCT: improvements in gait (stride length and cadence), with gains maintained but slightly diminished over time.
Strouwen et al., 2017<br>[62]	Idiopathic PD (UKBBC)<br>H&Y II–III<br>Ability to walk continuously for 10 minutes independently<br>MMSE ≥ 24<br>On stable medication<br>No significant hearing or vision impairments<br>No implanted DBS device or recent changes in its settings	Total: 121<br>CCT: 65 (Consecutive Task Training)<br>IDT: 56 (Integrated Dual Task Training)	Total: 65.93 ± 9.22<br>CCT: 66.05 ± 9.30<br>IDT: 65.80 ± 9.19	Total: 88/33 (M/F)<br>CCT: 49/16 (M/F)<br>IDT: 39/17 (M/F)	See above (Geroin et al., 2018)	See above (Geroin et al., 2018)	Walking speed under dual-task conditions (Stroop Test, mobile phone use, digit span),<br>Walking speed under single-task conditions<br>Recording of falls	Test 1: Before the start<br>Test 2: 6 weeks after the end of intervention (control baseline)<br>Test 3: Immediately after the 6-week training<br>Test 4: 12 weeks after the end of training (follow-up)	CCT & IDT: similar effects on gait, with short-term improvements in walking speed maintained at 12 weeks after the end of the training
					Virtual Reality				
Mirelman et al., 2016<br>[33]	Independent walking for 5 minutes<br>On stable medication<br>Two or more falls in the past 6 months	Total: 302<br>VR-TT (Intervention): 154 (PD = 66)<br>TT (Control): 148 (PD = 64)	VR-TT: 74.2 (6.9)<br>TT: 73.3 (6.4)	VR- TT: 74.2 (6.9) (M)<br>TT: 73.3 (6.4) (M)	VR-TT: obstacle courses, multiple pathways, distractions and real-life simulations with visual and auditory feedback.	TT: Similar program without virtual reality.	SPPB<br>Gait parameters (IMUs)<br>2MWT<br>Fall diaries	T0: Baseline<br>T1: 1 week after the end of intervention<br>T2: 1 month after the end of intervention	VR-TT: reduction in falls at 6 months post-training and a lower fall rate compared to the TT at the same point. Improvements were also

	Mild cognitive impairment (MMSE ≥ 21)<br>H&Y II–III				The program lasted over 6 weeks (x3/week/45 min)			T3: 6 months after the intervention	observed in gait variability, step height and walking speed.
Pelosin et al., 2022 [51]	Idiopathic PD (UKBBC)<br>H&Y II–III<br>Independent walking<br>On stable medication<br>Two or more falls in the previous six months	Total: 77<br>6-week intervention: 53<br>12-week intervention: 24	6 weeks: 73.84 ± 6.39<br>12 weeks: 74.09 ± 4.96	6 weeks: 20/31 (F/M)<br>12 weeks: 9/12 (F/M)	Two groups underwent treadmill and VR training under usual, dual-task, and obstacles conditions with progressive difficulty and visual/auditory feedback.<br>First group: 6 weeks, 18 sessions, 3 times/week, 45 minutes.<br>Second group: 12 weeks, 32 sessions, 3 times/week, 45 minutes.	None	Gait parameters (micro-accelerometers)<br>fall diaries<br>FES-I	FU-1m: 1 month after the end of the intervention<br>FU-6m: 6 months after the end of the intervention	Both groups improved gait, maintained at 6 months. The 12-week group showed greater gains in fall frequency, fear of falling, with all maintained.
Pelosin et al., 2020 [52]	Idiopathic PD (UKBBC)<br>2 or more falls in the 6 months before<br>Age 60- 85<br>Able to walk for 5 minutes unassisted<br>Hoehn & Yahr II-III<br>On stable medication<br>Excluded: dementia or MMSE <24, psychiatric co-morbidity and history of psychiatric treatment, stroke or other neurologic disorders, contra-indications to transcranial magnetic stimulation, anticholinergic or acetylcholinesterase inhibitors medications.	Treadmill Training (TT) n=22 [14 PD-8OA (Older Adults)]<br>Treadmill Training+ Virtual Reality (TT+VR) n=17 (10 PD-7OA)	TT: 71.9 (4.1)<br>TT+VR: 73.2 (3.6)	TT: 7/15 (M/F)<br>TT+VR: 6/11 (M/F)	See above [Mirelman et al, 2016]	See above [Mirelman et al, 2016]	Gait parameters (usual walking and obstacle negotiation)<br>Falls<br>MDS-UPDRS III	1 week before training (PRE)<br>1 week after training (POST)<br>6 months after training (FU)	PD patients had more falls than OA. TT+VR training reduced falls and improved gait during obstacle negotiation, increasing step length and reducing variability, with some effects maintained at FU (crossing step length), while TT showed no benefit.
Cueing Strategies									

Capato et al., 2020 [25]	See above under balance/gait training								
De Icco et al., 2015 [53]	Idiopathic PD (UKBBC)<br>Hoehn & Yahr II–IV<br>MMSE > 23<br>No significant comorbidities or recent hospitalization<br>On stable medication	Total: 46<br>Auditory group: 11<br>Visual group: 11<br>Control group: 24	Total: 74.4 ± 7.1 years<br>Auditory group: 78.1 ± 6.1<br>Visual group: 73.2 ± 6.9<br>Control group: 72.1 ± 7.3	Total: 24 (52% M)<br>Auditory group: 4/7 (F/M)<br>Visual group: 6/5 (F/M)<br>Control group: 12/12 (F/M	Two groups (auditory cues, visual cues) 40-minute general rehabilitation program and 20 minutes of gait training.<br>The auditory group used a metronome, while the visual group used colored floor lines.<br>4 weeks/ 5 sessions per week for 60 minutes.	Gait training without the use of external cues.	Spatiotemporal gait parameters (gait kinematic analysis)<br>UPDRS-III<br>FIM	T0: At baseline<br>T1: After 4 weeks<br>T2: 3 months after the end	Auditory cues increased step length and stride time, while visual cues improved swing phase but reduced speed. Both interventions improved gait after 4 weeks versus control, but were lost at 3 months. UPDRS-III and FIM improved immediately but not long-term.
Murgia et al., 2018 [54]	PD (UKBBC)<br>Hoehn & Yahr 1.5–3<br>MMSE > 24<br>FAB > 13<br>No significant auditory or psychiatric conditions<br>Ability to walk independently<br>No recent participation in a rehabilitation program<br>Treated with L-DOPA or dopaminergic agonists	Total: 38 (32 completed)<br>Ecological RAS (e-RAS): 16<br>Artificial RAS (a-RAS): 16	Total: 68.2 ± 10.5<br>e-RAS: 66.5 ± 10.9<br>a-RAS: 69.9 ± 10.1	Not reported	Two groups: ecological RAS, using participants' own step recordings and artificial RAS, using a fixed metronome tempo.<br>Both programs combined mobility, balance and posture exercises with 20 minutes of RAS gait training.<br>After 5 weeks (2 sessions/ week, 45 minutes), participants continued at home for 30 minutes, with an additional 12 weeks of home practice.	None	Tinetti Test<br>SPPB<br>STS<br>ABC Scale<br>Spatiotemporal gait parameters (8-camera Smart-D motion system)<br>4MWT<br>UPDRS-III<br>FIM<br>FES<br>FOGQ	T0: before the start<br>T5: end of the 5-week intervention<br>T17: 3 months after (17 weeks)	Both groups showed long-term improvements in the Tinetti Test, FES, UPDRS-III, SPPB and spatiotemporal gait parameters, except for gait cadence. The FOG index improved but was not maintained. Stronger effects were observed in the ecological RAS group.
Morris et al., 2009 [63]	Idiopathic PD<br>Hoehn & Yahr II- III<br>Able to walk 10m three times without assistance<br>MMSE > 23<br>Excluded: neurological	Total Sample for Randomization (n=28)<br>Group A (Strategies) (n=14)<br>Group B (Exercises)	In the final sample the ages ranged from 52 to 79 years<br>Group A<br>Mean age: 68	Not reported	Movement strategy training and musculoskeletal exercises daily.<br>Group A<br>14 sessions and mean total therapy time per person 526 minutes	Group B<br>13 sessions and mean total therapy time per person 461 minutes.<br>Conventional musculoskeletal	TUG<br>2MWT Balance pull test<br>10MWT<br>UPDRS	Baseline (admission)<br>After 2 weeks (discharge)<br>3 Month follow-up	Group A showed significant UPDRS and walking improvements from admission to discharge. 10MWT gains were maintained at 3 months, while 2MWT

	conditions, musculoskeletal, visual, or cardiopulmonary conditions that affected mobility, cognitive impairment, were not in hospital for 2 weeks	(n=14)<br>Lost to follow-up (n=2)	years<br>Group B<br>Mean age: 66 years		Cognitive strategies: external cues to enhance walking, turning, standing up from a chair and obstacle negotiation	exercises aimed to improve strength, ROM, posture, general fitness and function.			improvements regressed at follow-up. The exercise group showed no changes.
Martin et al., 2015 [55]	PD<br>Age > 65<br>FOG examined by NFOGQ<br>Independently mobile with or without walking aid<br>Stable medication<br>Exclusion: MMSE <24, comorbidities, unable to press metronome buttons or hear	Total: 21<br>Immediate- start (IS): 12<br>6-month delayed-start (DS): 9	Total: 72 (5.3)<br>IS: 72 (5.1)<br>DS: 72 (5.8)	Total: 8 (38)(F)<br>IS: 5 (42)(F)<br>DS: 3 (33)	Cued Up! Program. A 6-month home-based exercise and education program, using a metronome, similar to the Otago Program. It included 6 home visits, within the first 4 weeks, followed by weekly phone calls.<br>Therapy sessions (30 and 60 minutes) focused on metronome-paced exercises, exercise progression and education on FOG.<br>Participants were encouraged to continue their usual exercise.	The DS group began the intervention after a 6month wait period.	NFOGQ<br>Falls daily diary	Baseline<br>After 6 months (end of the IS intervention and DS end of the wait-list period)<br>After 12 months (IS group measured a further 6 months after the RAC intervention finished and the DS group at the end of their 6-month intervention)	Participants' attitudes about falling were mixed after the program, (worry less, more or no change)<br>Most (73%) perceived they were falling less frequently after the program, though NFOGQ scores did not change significantly.<br>All participants fell (at least twice) during the study and fall rates did not differ significantly between groups after.
Technology/ Robot Assisted Training									
Carda et al., 2012 [34]	Idiopathic PD (UKBBC)<br>Age <75 years<br>Hoehn & Yahr < III<br>No motor fluctuations<br>Independent walking ability<br>No prior specific gait training in the last 6 months	Total: 30<br>Lokomat: 15<br>TT: 15<br>Final participants: 28 (due to dropouts)	Total: 67.89 ± 5.4 years<br>Intervention group: 67.87 ± 7.05 years<br>Control group: 66.93 ± 5.13 years	Total: 60.7% M (n = 17)	Robotic-assisted body weight- supported treadmill training (Lokomat), with 50% and 30% support for 15 minutes each, for over 4 weeks (3 sessions/ week, 30 minutes), with treadmill speed progressively increased based on endurance.	Conventional treadmill gait training, with verbal guidance and immediate feedback.	TUG<br>10MWT 6MWT<br>UPDRS-III	Before the intervention,<br>Immediately after the intervention (4 weeks)<br>3 months post-intervention<br>6 months post-intervention.	Both groups improved in the 6MWT, 10MWT, TUG and UPDRS-III, with no between-group differences and gains were maintained at 6 months. The Lokomat group showed the greatest improvement at 3 months, with a slight decline at 6 months.
Furnari et al., 2017 [35]	Idiopathic PD (UKBBC)<br>Hoehn & Yahr II–III<br>On stable medication	Total: 38<br>EG = 19 (intervention group)	Total: 74.6 ± 10.2<br>EG: 71.5 ±	Total: 17 (44.7% F)	Robotic- assisted gait training (Lokomat) with progressive increases in speed and	Conventional physiotherapy	Tinetti Balance<br>Tinetti Walking,<br>10MWT	T0: before the start of treatment	Long-term improvements in balance and

	Independent walking ability<br>MMSE ≥ 23<br>No motor fluctuations<br>No significant cardiovascular, respiratory or other conditions affecting gait	CG = 19 (control group)	11.7<br>CG: 77.7 ± 8.3	EG: 8 (42.0%)<br>CG: 9 (47.3%)	reduced body weight support, combined with a 30-minute conventional physiotherapy program. The intervention lasted 4 weeks/ 12 sessions, 3 times per week.	and proprioceptive neuromuscular facilitation (PNF) techniques, for over 4 weeks (6 times/ week, 1 hour per session).	UPDRS-III	T1: immediately after 4 weeks of treatment<br>T2: 3 months after the end of treatment	gait were largely maintained, with a slight decline in both groups. However,only the EG retained improvements in the Tinetti Walking and UPDRS-III.
Picelli et al., 2013 [21]					See above under aerobic training				
Shen & Mak, 2014 [64]	Idiopathic PD<br>On stable medication<br>Independent walking for at least 10 meters<br>MMSE > 23<br>Hoehn and Yahr stage II–III	Total: 51<br>BAL group: 26<br>CON group: 25	BAL: 63.3 ± 8.0<br>CON: 65.3 ± 8.5	BAL group: 13/9 (M/F)<br>CON group: 12/11 (M/F)	12-week technology-assisted gait and balance program (KSD dance system and Smart EquiTest), involving voluntary multidirectional stepping with progressive difficulty.<br>Two 4-week supervised laboratory phases (3 sessions/week) followed by 4 weeks of home practice (20 minutes, 5 times/week).	Strength exercises (gym equipment), followed by rowing and gait exercises.	SLS<br>ABC Scale<br>Balance parameters (Smart EquiTest Balance Master KSD Technology)<br>Spatiometers gait parameters (GAITRite)<br>Falls	Baseline: at the start<br>End of intervention (Post): 3 months<br>Post3m: 3 months after the end of the intervention<br>Post12m: 12 months after the end of the intervention (i.e., a total of 15 months from baseline)	The BAL group showed sustained improvement in the ABC Scale and single-leg support at 3 and 12 months post-intervention, while both groups increased walking speed. Only the BAL group showed consistent improvements in step length across all assessments..
Shen & Mak, 2015 [65]	Idiopathic PD<br>On stable medication<br>Independent walking for at least 10 meters<br>MMSE > 23<br>Hoehn & Yahr II–III	Total: 51<br>BAL group: 26<br>CON group: 25	BAL: 63.3 ± 8.0<br>CON: 65.3 ± 8.5	BAL group: 13 (59% M)<br>CON group: 12 (52% M)	See above (Shen & Mak, 2014)	See above (Shen & Mak, 2014)	SLS<br>Spatiotemporal gait parameters (GAITRite)<br>Falls	Baseline: at the start<br>Post3m: after the 3rd month<br>Post6m: after the 6th month<br>Post12–15m: after the 12th–15th month	The BAL group had fewer fallers and sustained improvements in balance, gait and single-leg support at 3-, 6- and 15-months.
Miyai et al., 2002 [56]	PD<br>Hoehn & Yahr 2.5–3<br>No dementia: MMSE ≥ 27<br>Presence of freezing, but no "on–off" phenomenon<br>On stable medication	Total: 24<br>BWSTT group: 11<br>Conventional Physiotherapy (PT) group: 9	BWSTT: 69.5 ± 1.9<br>PT: 69.8 ± 1.5	Total: 50% M (12/24)<br>BWSTT group: 5/6 (M/F)<br>PT group: 5/4 (M/F)	Body weight-supported treadmill with decreasing body-weight support and progressive speed, plus 45-	Conventional physiotherapy program and occupational therapy.	10MWT<br>UPDRS	Baseline<br>Follow-ups: 1-, 2-, 3-, 4-, 5-, and 6-months post-intervention	The BWSTT group showed greater improvements in walking speed, 10MWT, stride length and step count at 1–4 months compared

					minute occupational therapy (3 times/ week for one month).				to the PT group, with no significant change in UPDRS.
Dance									
Rawson et al., 2019 [46]	See above under aerobic training								
Pohl et al., 2020 [36]	Age <18 years<br>PD<br>Hoehn & Yahr ≤ 3<br>On stable medication<br>Ability to walk 10 meters without assistance<br>MoCA ≤ 25	Total: 46<br>Intervention group: 26<br>Control group: 20	Intervention group: 69.7 ± 7.0 years<br>Control group: 70.4 ± 6.0 years	Intervention group: 19 M (73%)<br>Control group: 13 M (65%)	The 12-week Ronnie Gardiner program included 60-minute sessions (2/week) with 50 minutes of choreo-motor exercises.	Usual care	TUG Mini-BESTest<br>FOGQ<br>FES-I	Baseline<br>2 weeks after the end of the intervention<br>3 months after the end of the intervention	The intervention improved FES-I at 3 months, but not Mini-BESTest or TUG dual-tas.Short-term gains were not maintained.
Yoga									
Cheung et al., 2018 [66]	Hoehn & Yahr I–III<br>Age 45–75<br>On stable dopaminergic medication<br>Ability to walk 6 meters with or without an assistive device<br>Absence of atypical Parkinson's forms<br>No serious comorbidities<br>MoCA<26<br>No regular yoga practice prior to the study	Total: 20<br>Yoga group: 10<br>Control group (CG): 10	Total: 63 ± 8 years<br>Yoga group: 63.5 ± 8.5 years<br>Control group (CG): 65.8 ± 6.6 years	Not reported	Yoga program for 12 weeks. It included 2 sessions/ week, 60 minutes.	No therapy during the study but joined the yoga program afterward.	mUPDRS-III	At baseline<br>After 12 weeks of intervention<br>6 months after the end of the intervention (follow-up)	The yoga group showed improvements in motor function, physical activity, but were not maintained long-term. mUPDRS-III improved immediately, with no data reported on long-term maintenance.
Kwok et al., 2019 [67]	Idiopathic PD<br>Hoehn & Yahr stage I–III<br>Age >18 years<br>Ability to stand or walk with or without an assistive device	Total: 138<br>Yoga group: 71<br>Control group (CG): 67	Total: 63.6 ± 8.7 years<br>Yoga group: 63.7 ± 8.2 years<br>Control group (CG): 63.5 ± 9.3 years	Total: 65 (47.1) (M)<br>Yoga: 37 (52.1) (M)<br>CG: 28 (41.8) (M)	MY-PD yoga program for 8 weeks with one 90-minute group session per week and 20 minutes of individual practice twice weekly.	Stretching and resistance exercise program.	TUG<br>MDS-UPDRS-III	Baseline (T0)<br>Immediately after the 8-week intervention (T1)<br>Follow-up 3 months later (T2, i.e., 20 weeks after T0)	Moderate improvements in MDS-UPDRS-III immediately post-intervention and at 3 months, while no changes were seen in TUG.

Tai Chi/ Qigong									
Gao et al., 2014 [68]	Idiopathic PD<br>Independent walking<br>Recorded ≥1 fall in the past 12 months<br>Age >40 years<br>MMSE ≥ 24<br>Ability to perform moderate-intensity exercise for 60 minutes	Total: 76<br>Tai Chi group: 37<br>Control group (CG): 39	Tai Chi: 69.54 ± 7.32<br>CG: 68.28 ± 8.53	Tai Chi: 23 (62.16% M)<br>CG: 27 (69.23% M)	24-form Yang style Tai Chi and<br>usual medical treatment for 12 weeks/ 32 sessions of 60 minutes.	Usual medical treatment	BBS<br>UPDRS-III<br>TUG<br>Falls diary	At baseline<br>12 weeks after the end of the intervention<br>6 months after the intervention	Reduced number of falls in the Tai Chi group at 6 months after the end of the intervention (only falls assessed at follow-up)
Li et al., 2012 [24]	See above under resistance training								
Li et al., 2014 [69]	PD<br>Age 40–85 years<br>Hoehn & Yahr stage I–IV	Total: 195<br>Tai Chi group: 65<br>Resistance training (RES) group: 65<br>Stretching (control) group: 65	See above (Li et al., 2012)	See above (Li et al., 2012)	See above (Li et al., 2012)	See above (Li et al., 2012)	50- foot speed walk test<br>UPDRS- ME	As in the study Li et al., 2012	Tai Chi improvements in patient- reported outcomes correlated with UPDRS- ME and 50-foot walk scores and participants reporting benefits were more likely to continue exercising.
Schmitz-Hübsch et al., 2006 [37]	PD regardless of stage and motor complications<br>On stable medication<br>MMSE > 24<br>Excluded: prior Qigong experience	Total: 56<br>Qigong group: 32<br>Control group (CG): 24	Qigong group: 64 ± 8<br>Control group (CG): 63 ± 8	Qigong: 24/8 (M/F)<br>CG: 19/5 (M/F)	Qigong program ("Frolic of the Crane" and "Eight Brocades"), seated or standing and home practice. It included 16 lessons over two 8-week cycles, with one 60-minute session/week.	No other intervention.	UPDRS-III	Baseline<br>3 months after the start<br>6 months after the start<br>12 months after the start of the intervention	Qigong group showed significant improvement in UPDRS-III (question 30) at 3 and 6 months, with partial maintenance at 12 months.
Music Therapy									
Pohl et al., 2020 [36]	See above under Dance								
Pilates									
Coban et al., 2025 [38]	PD<br>Hoehn & Yahr stage II–III<br>Age >45 years<br>MMSE ≥ 24	Total: 32<br>ParkinsonPilates (PP) group: 18<br>Conventional	PP: 70.19 ± 8.88<br>CP: 72.0 ± 7.3	PP: 5 (31.25% M)<br>CP: 9 (56.25% M)	A 12-week ParkinsonPilates program using multisensory cues, with 24 supervised sessions (2 per week,	Active conventional physiotherapy program (strengthening,	GABS<br>NFRT<br>BBS<br>TUG	Baseline<br>6th week (mid-intervention)<br>12th week (end of	The experimental group showed lasting improvements in BBS, FRT, GABS, NFRT and

	Turkish language proficiency<br>Diagnosis ≥ 2 years<br>No other neurological conditions or medication changes during the study	Physiotherapy (CP) group: 18			60 minutes each) and 4 home practice sessions per week.	flexibility, endurance exercises).	FRT<br>UPDRS-III	intervention)<br>24th week (3-month follow-up)	gait rhythm at 3 months, while TUG and UPDRS-III were not maintained.
Multimodal Training/ Physical Therapy									
Ashburn et al., 2007 [39]	Idiopathic PD<br>Hoehn & Yahr 2-4<br>Independently mobile<br>Living at home in the community<br>>1 fall in the previous 12 months	Total: 142<br>EG: 70<br>CG: 72	EG: 72.7 (9.6)<br>CG: 71.6 (8.8)	EG: 38 (54% M)<br>CG: 48 (67% M)	Strength, ROM exercises, balance training, strategies for fall prevention and movement initiation and compensation technique, daily for one- hour at home for 6 wk. After the end, monthly follow- up phone calls were conducted to encourage their training.	Usual care	TUG<br>BBT<br>FRT<br>Chair Stand Test<br>SAS<br>Falls diaries	Baseline<br>8 weeks<br>6mo (18 weeks after the end of the program)	The exercise group showed lower fall and injury rates at 8 weeks and 6 months, especially in less severe participants and improved FR at 6 months.<br>No significant differences were observed in BBT, SAS, TUG, ROM, or muscle strength.
Dipasquale et al., 2017 [40]	Idiopathic PD<br>Diagnosis ≥ 24 months<br>Hoehn & Yahr II<br>Medical therapy unchanged<br>Ability to follow the study protocol	Physiotherapy Group (Phys): 20<br>General exercise (GE): 20<br>Totally, 9 drop outs (4 Phys and 5 GE)	Phys: 69.9 (6.42)<br>GE: 66.4 (9.32)	Phys: 13 (65% M)<br>GE: 13 (65% M)	The physiotherapy group followed the Royal Dutch Society for Physical Therapy guidelines (transfers, balance, gait, posture). Two weekly sessions of one hour each for four months.	General exercise program recommended for patients with PD.	FIM<br>TUG<br>UPDRS-III	T0: Before the start of the treatment<br>T1: At the end<br>T2: Follow- up 135 ± 20 days after the end of the treatment	The physiotherapy group showed sustained FIM improvements, decreased UPDRS-III and better TUG and walk performance.<br>The GE group had short-term FIM gains, increasing UPDRS-III and declined mobility over time.
Ellis et al., 2005 [70]	Idiopathic PD<br>On stable medication<br>Hoehn & Yahr II- III<br>At least 1 score of 2 or more for at least 1 limb for either the tremor, rigidity, or bradykinesia item of the UPDRS<br>Walk independently	Total: 68<br>Group A: 35<br>Group B: 33<br>63 completed the intervention	Total: 64 ± 8.6<br>Group A: 64 ± 8.4<br>Group B: 63 ± 8.8	Total: 51/17 (M/F)<br>Group A: 25/10 (M/F)<br>Group B: 26/7 (M/F)	Group A: 6-week physiotherapy program plus medication, followed by 6-week of medication only.<br>Group B had a control period first, then 6 weeks physiotherapy. Sessions were 1.5 hours, twice a week.	Group A and B, when on control phase	Comfortable Walking Speed (CWS)<br>UPDRS III	t0: Baseline<br>t6: after the initial 6 weeks<br>t12: after the second 6-week period<br>t24: 3 months after the second 6-week period	Group A showed short-term motor improvements (CWS) after the initial 6 weeks, but CWS gains persisted through 12 weeks.<br>Long-term improvements were not main-

	Age 35- 75 years<br>MMSE ≥ 24<br>No other severe medical conditions<br>No participation on other rehabilitation program								tained. Group B improved CWS after their intervention, with significant gains at 24 weeks.
Zanchet et al., 2025 [41]	PD Diagnosis<br>Age 18- 80<br>On stable medication or DBS with stable stimulation parameters<br>No severe PD or conditions limiting exercise (excluded patients presenting a Hoehn and Yahr scale >3)	Total: 44<br>APA+ (Adapted Physical Activity): 22<br>APA-: 22	Total: 67.7 ± 7.3<br>APA-: 68.1 ± 7.3<br>APA+: 67.3 ± 7.5	Total: 30 (68.2) (M%)<br>APA-: 15 (68.2) (M%)<br>APA+: 15 (68.2) (M%)	3-month APA program, with 2 sessions/week for 1 hour. Aerobic, strength and stretching exercises, plus physical activities, education and a 2-weekly physiotherapy focusing on gait, balance.	The APA- group could engage in other physical activities, as mentioned for the APA+ group.	UPDRS -III, 6MWT	Baseline<br>M3: Immediately after the end of the 3-month intervention<br>M9: 6 months after the end of the program	The UPDRS-III scores decreased in APA+ group and increased in the APA- group by M3, with no difference at M9.
Mak et al., 2024 [13]	Age 30–80 years<br>Idiopathic PD<br>Modified Hoehn & Yahr 1.5–3<br>Ability to walk ≥ 30 meters independently<br>No "on–off" motor fluctuations<br>MoCA ≤ 25 (indicating reduced cognitive function)	Total: 99<br>Balance & Brisk Walking (B&B) group: 49<br>Control group (CG): 50	B&B: 64/11<br>CG: 62/10	B&B: 26/23 (F/M)<br>CG: 33/17 (F/M)	12-month, group- based program combining balance,brisk walking, music and dual- task activities. The first 6 weeks, they trained 6 times/week. From weeks 7-26, 4 times (90 minutes) and from 27-52, up to 180 minutes/week and home practice 2-3 times weekly.	Flexibility, strengthening and stretching exercises for 180 minutes per week..	TUG Dual Task<br>Mini-BESTest<br>ABC Scale<br>Comfortable Gait Speed (CGS)<br>6MWT<br>MDS–UPDRS III<br>Falls diary	PRE: before and at the start of the intervention<br>POST: at 6 months (end of intervention)<br>FU: at 12 months (6 months after the end of the intervention)	The B&B group showed lasting improvements in the MDS-UPDRS-III,Mini-BESTest, 6MWT and Dual Task performance, while the ABC Scale remained stable in the B&B group.
Frazzitta et al., 2012 [71]	Idiopathic PD<br>Hoehn & Yahr 3<br>Walk without physical assistance<br>MMSE ≥ 26<br>No comorbidity<br>No vestibular/ visual dysfunction limiting locomotion or balance<br>On stable medication	Group 1 (treated patients- IRT): 25<br>Group 2 (control): 25	Group 1: 72 ± 7<br>Group 2: 70 ± 7	Group 1: 11/14 (M/F)<br>Group 2: 13/12 (M/F)	Intensive rehabilitation program (IRT) for 4 weeks, consisting of 3 daily sessions, 5 days a week (balance, gait training with cues and occupational therapy).<br>After discharge, they continued exercises and walked for 30 minutes daily.	Pharmacological treatment and general instructions for home exercise.	UPDRS- III<br>UPDRS Total	T0: First admission<br>T1: First discharge (4 weeks)<br>T2: Second admission (after 11 months)<br>T3: Second discharge (after the end of the 2nd cycle of treatment)	In the IRT group, UPDRS scores were maintained, with improvements seen in both treatment cycles, while the control group showed progressive deterioration.

Frazzitta et al., 2015 [42]	Idiopathic PD<br>Hoehn & Yahr 1- 1.5<br>Walk without physical assistance<br>MMSE ≥ 26<br>No serious comorbidity<br>No vestibular/ visual dysfunction limiting locomotion or balance	Total: 40<br>MIRT (Multidisciplinary Intensive Rehabilitation Treatment) Group: 20<br>Control Group: 20<br>Totally, 9 drop out (4 MIRT and 5 CG)	MIRT Group: 69 ± 6<br>Control Group: 68 ± 8	MIRT Group: 45 (% M)<br>Control Group: 60 (% M)	4-week multidisciplinary program with 3 1-hour daily sessions, 5 days/ week (balance, gait training with cues and occupational therapy), with encouragement to continue exercises at home.	Pharmacological treatment	TUG 6MWT UPDRS III	T0: Baseline<br>T1: 6 months<br>T2: 1 year<br>T3: 18 months<br>T4: 24 months	The MIRT group showed improvements immediately after, with sustained gains in UPDRS III, TUG at T1, T3 and T4.
Palamara et al., 2017 [72]	Diagnosis of PD<br>Hoehn & Yahr 2.5-3<br>Stable medication for the last 8 weeks and during the hospitalization<br>MMSE ≥ 24	Total Sample (n=34)<br>Group A: Multidisciplinary Intensive Rehabilitation Treatment (MIRT) (n=17)<br>Group B: Multidisciplinary Intensive Rehabilitation Treatment Associated with Aquatic Therapy (MIRT-AT) (n=17)	MIRT Mean age=70.8±5.3<br>MIRT-AT Mean age=70.9±5.7	Total males: 20<br>Total females: 14<br>MIRT Males: 11 Females: 6<br>MIRT-AT Males: 9 Females: 8	MIRT: 4-week program in hospital - 4 daily sessions of physical therapy for five days and one hour of physical exercise on the sixth day (e.g. aerobic exercises, gait with cues, occupational and speech therapy, robotic- assisted- virtual reality).	MRIT-AT: Same as MIRT + 3 sessions/week of aquatic therapy (aerobic exercises and physical activities).	BBS UPDRS TUG	After the 4-week intervention period<br>6-month follow-up	No significant changes were observed immediately or at the 6 months, though water features may have helped maintain BBS improvement at follow-up.
Avenali et al., 2021 [43]	PD (UKBBC)<br>Hoehn & Yahr ≤ 3<br>PD-MCI single- or multiple- domain (level II)<br>No other psychiatric, neurological, or severe pathological conditions<br>On stable medication	Total: 34 (firstly 40)<br>PT (Physical Therapy): 15<br>CT (Control group): 19	Total: 72.3 ± 6.5<br>PT: 73.2 ± 7.1<br>CT: 71.6 ± 6.0	Total: 21/13 (M/F)<br>PT: 7/8 (M/F)<br>CT: 14/5 (M/F)	Physiotherapy program: aerobic exercises, coordination exercises, treadmill training and cognitive engagement exercises.<br>6 sessions/ 60 minutes for 4 weeks.	Drug treatment and advice to remain physically active.	Tinetti balance and gait score MDS-UPDRS III<br>Hauser Index	T0: baseline<br>T1: 4 weeks after baseline<br>T2: 6 months after baseline (5 months after the end of the treatment)	PT improved motor performance at T2. UPDRS-III and Tinetti scores declined from T1 to T2, but improved from T0 to T2, with the intervention group showing greater UPDRS-III.
Monticone et	Idiopathic PD<br>Hoehn & Yahr 2.5- 4<br>A decline in function	Experimental Group (EG): 35	EG: 74.1 (6.0)<br>CG: 73.4 (7.0)	EG: 24/11 (M/F)<br>CG: 22/13 (M/F)	Motor training (transfers, functional activities, treadmill training, balance and	Neuromotor training (joint mobilization,	BBS<br>MD- UPDRS III<br>FIM	Before treatment<br>Post- treatment: 8 weeks later	Both groups improved motor function, but EG showed greater and

al., 2015 [44]	Age >50 years<br>Disease duration >10 years<br>On stable medication<br>No other severe medical conditions	Control Group (CG): 35			gait exercises etc.), cognitive and ergonomic training for over 8 weeks, with daily 90-minute sessions.	strength exercises, stretching, balance and gait training) for 8 weeks.		1-y follow-up: 12 months after discharge	sustained gains, with larger improvements in BBS and FIM over time.
**LSVT BIG**									
Dashtipour et al., 2015 [45]	PD diagnosis (UKBBC)<br>30-90 years<br>On stable medication<br>No other medical conditions	General Exercise (GE): 5<br>LSVT BIG: 6	Total: 63.4<br>GE: 64.0 (4.2)<br>LSVT BIG: 62.8 (13.9)	F/M ratio: 6/5	LSVT BIG: large amplitude functional movements performed for 60 minutes over a period of 4 weeks.	General exercise protocol: 30 minutes treadmill training and seated upper limb exercise.	UPDRS	Baseline<br>Immediately after the end of the program<br>After 3 and 6 months	The UPDRS scores showed significant improvement at 6 months. Both interventions were equally effective at the 6- month follow-up.

Abbreviations: UK PDS BBC= UK Parkinson's Disease Society Brain Bank Criteria; PD= Parkinson's Disease; MMSE= Mini Mental State Examination; FOG= Freezing of Gait; FOGQ= Freezing of Gait Questionnaire; BBS= Berg Balance Scale; BBT=Berg Balance Test; TUG= Timed Up and Go; 10MWT= 10-Meter Walk Test; FES= Falls Efficacy Scale; FES-I= Falls Efficacy Scale International; UPDRS-III= Unified Parkinson's Disease Rating Scale; ABC Scale: Activities-Specific Balance Confidence Scale; 6MWT= 6 Minute Walk Test; 4MWT= 4-Meter Walk Test; 2MWT= 2-Minute Walk Test; SPPB= Short Physical Performance Battery; STS= Sit-to-Stand Test; FTSTS= Five Times Sit-to-Stand; MDS–UPDRS-III= Movement Disorder's Society UPDRS-III; FDM-T=Force Distribution Measurement Treadmill; OLS= One-Leg Stand test; DGI= Dynamic Gait Index; FWD= Forward; BKD= Backward; PCI=Phase Coordination Index; EG=Experimental Group; CG=Control Group; F=Females; M=Males; SLS=Single Leg Stance; NFOGQ= New Freezing of Gait Questionnaire; PNF= Proprioceptive Neuromuscular Facilitation; PTFMB= Provocative Test for Freezing and Motor Blocks; GABS=Clinical Gait And Balance Scale; FRT or FR=Functional Reach Test; NFRT=Nelson Foot Reaction Test; SAS=Self-assessment Parkinson's Disease Disability Scale; FIM=Functional Independence Measure; Mini-BESTest=Mini-Balance Evaluation Systems Test; RAS= Rhythmic Auditory Stimulation; DBS= Deep Brain Stimulation; FU= Follow-up; LSVT=Lee Silverman Voice Treatment.

NeuroSci
