# Supplementary figures and images for: Sustained Effects of Physiotherapy Interventions on Balance, Gait, and General Motor Function in Patients with Parkinson’s Disease: A Systematic Review and Meta-Analysis
Source: NeuroSci. 2026 Apr 3;7(2):42. doi: 10.3390/neurosci7020042 (PMC13119462; doi:10.3390/neurosci7020042)

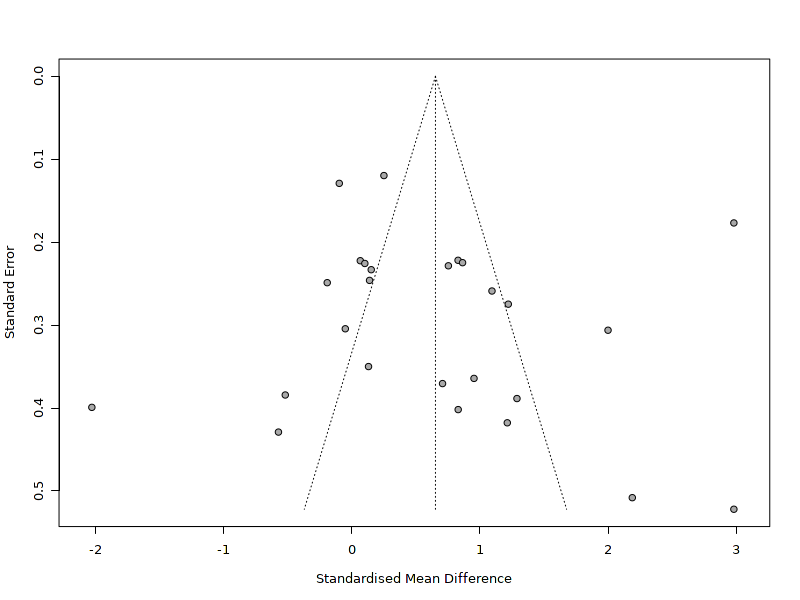

Supplement: Supplementary file 1 [file neurosci-07-00042-s001.zip › Figure S1.Funnel plot A.png]

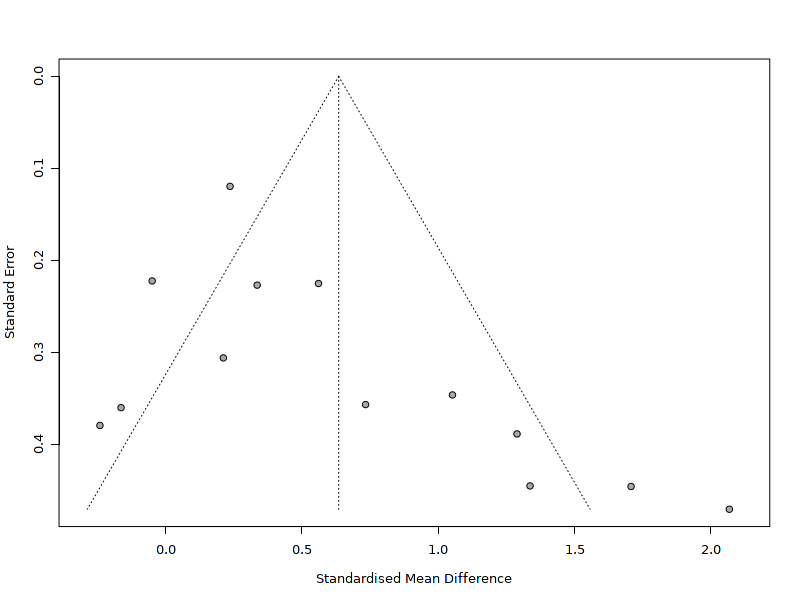

Supplement: Supplementary file 1 [file neurosci-07-00042-s001.zip › Figure S2.Funnel plot B.png]

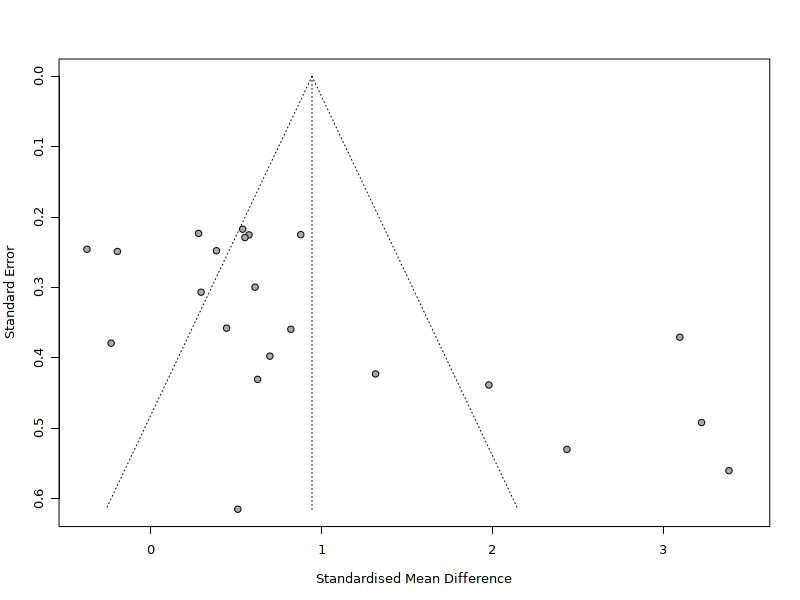

Supplement: Supplementary file 1 [file neurosci-07-00042-s001.zip › Figure S3.Funnel Plot C.png]
